# Association between drinking water quality and mental health and the modifying role of diet: a prospective cohort study

**DOI:** 10.1186/s12916-024-03269-3

**Published:** 2024-02-02

**Authors:** Shuduo Zhou, Mintao Su, Peng Shen, Zongming Yang, Pengfei Chai, Shengzhi Sun, Hongbo Lin, Liming Shui, Na Zhang, Ming Xu, Zhi-Jie Zheng, Jianbing Wang, Zhenyu Zhang, Kun Chen

**Affiliations:** 1 Department of Global Health, School of Public Health, Peking University, 38 Xue Yuan Road, Beijing, 100191 Haidian District China; 2 Institute for Global Health and Development, Peking University, Beijing, China; 3 Yinzhou District Center for Disease Control and Prevention, Ningbo, 315040 China; 4 Department of Public Health, and Department of National Clinical Research Center for Child Health, The Children’s Hospital, Zhejiang University School of Medicine, Hangzhou, 310058 China; 5 School of Public Health, Capital Medical University, Beijing, 100069 China; 6 Yinzhou District Health Bureau of Ningbo, Ningbo, 315040 China; 7 Department of Nutrition and Food Hygiene, School of Public Health, Peking University, Beijing, China; 8 Department of Epidemiology and Biostatistics, Zhejiang University School of Public Health, Hangzhou, 310058 China; 9 Department of Epidemiology and Biostatistics, National Clinical Research Center for Child Health of the Children’s Hospital, Zhejiang University School of Medicine, Hangzhou, 310058 China; 10 Institute of Carbon Neutrality, Peking University, Beijing, China; 11 Department of Epidemiology and Biostatistics, and Cancer Institute of the Second Affiliated Hospital, Zhejiang University School of Medicine, Hangzhou, 310058 China

**Keywords:** Depression, Anxiety, Metals element, Nonmetals element, Drinking water, Cohort study

## Abstract

**Background:**

Environmental factors play an important role in developing mental disorders. This study aimed to investigate the associations of metal and nonmetal elements in drinking water with the risk of depression and anxiety and to assess whether diets modulate these associations.

**Methods:**

We conducted a prospective cohort study including 24,285 participants free from depression and anxiety from the Yinzhou Cohort study in the 2016–2021 period. The exposures were measured by multiplying metal and nonmetal element concentrations in local pipeline terminal tap water samples and total daily drinking water intakes. Cox regression models adjusted for multi-level covariates were used to estimate adjusted hazard ratios (aHRs) and 95% confidence intervals (95%CIs).

**Results:**

During an average follow-up period of 4.72 and 4.68 years, 773 and 1334 cases of depression and anxiety were identified, respectively. A 1 standard deviation (SD) increase in manganese exposure reduced the incidence of depression by 8% (HR 0.92, 95%CI 0.88 to 0.97). In contrast, with a 1 SD increase in copper and cadmium exposure, the incidence of depression increased by 6% (HR 1.06, 95%CI 1.01 to 1.11) and 8% (HR 1.08, 95%CI 1.00 to 1.17), respectively. The incidence of anxiety increased by 39% (HR 1.39, 95%CI 1.20 to 1.62), 33% (HR 1.33, 95%CI 1.03 to 1.71), and 14% (HR 1.14, 95%CI 1.03 to 1.25) respectively for a 1 SD increase in manganese, iron, and selenium exposure. Diets have a moderating effect on the associations of metal and nonmetal elements with the risk of anxiety. Stronger associations were observed in older, low-income groups and low-education groups.

**Conclusions:**

We found significant associations between exposure to metal and nonmetal elements and depression and anxiety. Diets regulated the associations to some extent.

**Supplementary Information:**

The online version contains supplementary material available at 10.1186/s12916-024-03269-3.

## Background

Mental diseases are the leading causes of disability and premature death worldwide, accounting for approximately 16% of the global disability-adjusted life years (DALYs) in 2019 [1, 2]. The COVID-19 pandemic has amplified this burden, with an approximate 25% increase in global anxiety and depression prevalence [3, 4]. Given their prevalence throughout the human lifespan, the effective treatment and prevention of depression and anxiety present significant public health challenges [5, 6]. The incidence of depression and anxiety is influenced by a combination of factors such as genetics, social environment, and physical environment [7–9]. Identifying modifiable risk factors among these, particularly those related to social and environmental contexts, is crucial in developing effective preventive strategies [10].

Micro-elements, integral to biochemical functions, may also have a neurobiological influence [11]. A growing number of recent studies are delving into the role of micro-elements in the development of depression and anxiety [12, 13]. Existing research has revealed the relationships between heavy metals like cadmium and increased risk of depression and anxiety [14]. Moreover, elements such as manganese, copper, and selenium can function as antioxidants combating oxidative stress, a key player in depression’s pathophysiology [15]. Iron and zinc are also critical elements in regulating cellular function and neuromodulation and reduce the risk for depression [16, 17], while no significant associations were found in other studies [18, 19]. In summary, most previous research is cross-sectional, and the associations between metal and nonmetal elements and depression or anxiety remained controversial [20, 21]. High-quality epidemiological studies with prospective designs are needed to understand the environmental risk factors of depression and anxiety.

Previous studies showed that healthy eating habits, for example, a varied diet rich in fruits and vegetables, may reduce the risk of mental disorders [22, 23]. The content of compounds that are positive for mental health in fruits and vegetables may help reduce oxidative stress and inflammation [24]. While red meat intake may elevate levels of pro-inflammatory cytokines, which plays a potential role in the etiology of depression [25]. It is still unknown whether diet could modulate the association between long-term exposure to metal and nonmetal elements in drinking water and the risk of mental diseases.

Every adult consumes more than a liter of drinking water daily, with the metal and nonmetal elements it contains playing a significant role in their health [26]. Longitudinal studies that examine the relationship between these elements in drinking water and depression and anxiety are scarce in the Chinese population. As China faces rapid aging and modernization, a deeper understanding of this relationship becomes increasingly pertinent [27]. In this study, we aimed to estimate the association between long-term exposure to various elements (manganese, zinc, copper, iron, aluminum, cadmium, selenium, and fluorine) in drinking water and the risk of depression and anxiety in a prospective cohort from southeast China. We aim to investigate how diet moderates these relationships and identify the populations most susceptible to these risks.

## Methods

### Study population

**Fig. 1:**
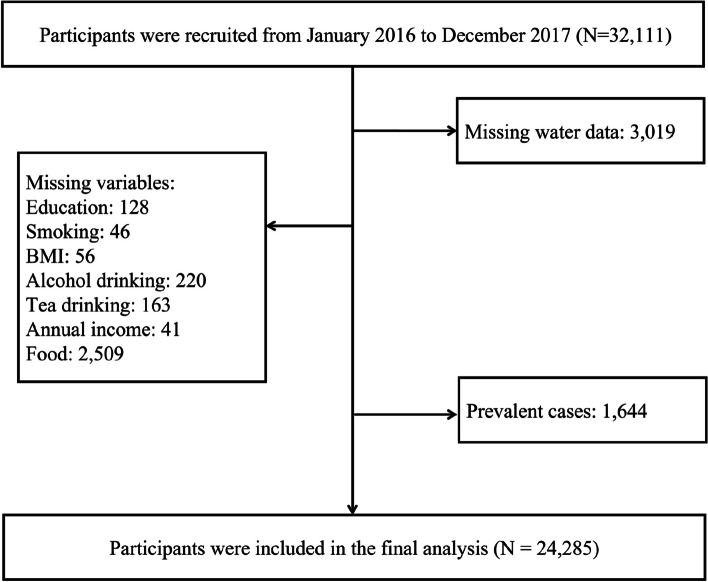
Flow diagram of the study population

The participants in our study were from the prospective Yinzhou Cohort study in Ningbo, a major port city in southeastern China with a population of more than 9.5 million in 2021. The design and data collection process has been widely published [28]. All permanent residents aged 18 and over from 9 townships in the Yinzhou district were invited to participate in the cohort from January 2016 to December 2017. The baseline cohort included 32,111 participants. We excluded participants with missing exposure data (*n* = 3019), missing important covariates at baseline (*n* = 654), missing food intake information (*n* = 2509), and pre-existing depression or anxiety (*n* = 1644). In our final analysis, 24,285 participants were included. We excluded the participants with anxiety in the follow-up (*n* = 1316) for depression analysis, and we excluded the participants with depression in the follow-up (*n* = 765) for anxiety analysis (Fig. 1). The Zhejiang University School of Medicine Ethics Committee approved this study (approval no. 2015-ZJU-023), and written informed consent was obtained from all participants before the baseline investigation.

### Outcome ascertainment

The Yinzhou Health Information System (YHIS), an information system that compiles all health service institutions’ data in Yinzhou District, records data from chronic disease monitoring, electronic medical records, death registry records, and routine health care records. From this system, we identified participants diagnosed with depression and anxiety through International Classification Disease 10 codes (F32, F41, respectively). Participants excluding prevalent depression or anxiety were followed from the time of their enrollment until they received a diagnosis of depression or anxiety, were lost to follow-up, follow-up ended (December 31, 2021), or died, whichever occurred first.

### Exposure assessment

We collected terminal tap water samples from 37 distinct sites in the Yinzhou district 4 times per year, once each season. The metals and nonmetals elements in tap water were originated from natural water resources. We used atomic absorption spectrophotometry to measure the metal and nonmetal elements in the tap water. We matched these samples to our participants using their residential addresses. Exposures were assigned to participants based on the location of terminal tap water samples and the participants’ residential addresses. Relying on the parameters from the Manual of Drinking Water Exposure Parameters for Populations in Typical Cities of Key Basins in China (adult volume), we calculated daily exposure to metal and nonmetal elements in drinking water. The calculation accounted for the total daily drinking water intake of adults, differentiated by gender and across 6 age strata [29].

1 $${{\text{Exposure}}}_{i}={C}_{i} \times \sum_{{\text{group}}=1}^{6}(\frac{{N}_{{\text{group}}}}{{\sum }_{j=1}^{6}{N}_{j}} \times {V}_{{\text{group}}})$$

In Eq. (1), ${{\text{Exposure}}}_{i}$ represents the metal and nonmetal elements exposure, ${C}_{i}$ represents the concentration of metal and nonmetal elements in drinking water, ${N}_{{\text{group}}}$ and ${N}_{j}$ represent the sample size of each age stratum in the manual data, and ${V}_{{\text{group}}}$ represents the total daily drinking water intake of each naive age stratum in the manual data.

To facilitate comparison of exposure test results, we calculated standardized *Z* scores scaling them to the exposure measurements by subtracting the mean of exposure measurement from each measurement test value at individual sites and dividing by the exposure measurement standard deviation (SD).

### Diets measurement

In the baseline survey, participants were asked “how often do you eat green leafy vegetables, fruits, meat (red and white), and marine products (seafood and freshwater) per week on average?” We obtained the weekly frequency of food intake from the questionnaire (almost every day [≥ 6 days/week], often [4–5 days/week], occasionally [≤ 3 days/week]). Participants were categorized as low, moderate, or high consumers depending on whether they responded occasionally, often, or almost every day, respectively.

### Covariates

The well-trained medical staff collected participant’s sociodemographic status: age (under 55, 55–65, or over 65), educational attainment (primary school and below or middle school and above), and annual household income (< $4000 versus ~ $4000), and lifestyle factors including body mass index (underweight, normal, overweight, or obesity), current smoking status (never, former, or current), alcohol consumption status (never, former, or current), current tea drinking status (never, former, or current), and medical history of stroke, diabetes, hypertension, cancer, and dyslipidemia (yes versus no) through a structured questionnaire. Body mass index (BMI) was calculated by dividing weight in kilograms by height in meters squared. The study defined current smokers as those who smoked at least one cigarette daily for more than a year or consumed five or more packs per month. Alcohol drinkers were classified as those who consumed at least 100 g of alcohol weekly. Lastly, tea consumers were defined as those who drank more than two cups of tea weekly for a period exceeding 2 months.

### Statistical analysis

Variables were summarized as *n* (%) for categorical variables and median (interquartile range (IQR)) for continuous variables. We employed Cox regression models to estimate the metal and nonmetal exposure in drinking water and the risk of depression and anxiety. The proportional hazards assumption was tested using Schoenfeld residuals.

We fitted three models to adjust for potential risk factors, based on a priori assumptions of the causal relationships, and tested by a directed acyclic graph (Additional file 1: Fig. S1). Model 1 was adjusted for age and educational attainment. Model 2 was further adjusted for BMI, annual household income, current smoking status, alcohol consumption status, and current tea-drinking status, which were potential risk factors and had been adjusted in previous studies. We also adjusted medical history of stroke, diabetes, hypertension, cancer, and dyslipidemia, which were considered as potential mediators. In model 3, we further adjusted the consumption of green leafy vegetables, fruits, meat, and marine products, which were considered to be possible mediators. Model 3 was suggested as the primary model in our study. Cluster-robust standard errors were used to account for clustering by water sources.

We investigated how diet moderates the associations between metals and nonmetals in drinking water and the risks of depression or anxiety. We used likelihood ratio tests to evaluate the effect modification by comparing models that included an interaction term between metals and nonmetals exposure and the effect modifier to models without the interaction term. We additionally ran subgroup analyses to identify the populations most susceptible to the risks of exposure to metal and nonmetal in drinking water and depression or anxiety by including interaction terms between exposures and age, annual household income, education, smoking status, alcohol consumption, tea consumption, and BMI. Stratum-specific HRs were calculated using the same interaction model’s appropriate coefficients and variance–covariance matrix.

We also conducted sensitivity analyses to demonstrate the robustness of the results. First, we excluded the events that occurred within the first years of follow-up to reduce potential reverse causation. Second, we calculated the *E* values to examine the degree to which unmeasured confounding potentially affected our findings [30, 31]. All associations were presented as HRs with corresponding 95%CIs. A 2-sided *P*-value < 0.05 was considered statistically significant. Stata version 16.0 for Mac (Stata Corp, College Station, TX, USA) and R Studio version 1.2.5042 (the R Project for Statistical Computing, Vienna, Austria) were used for the statistical analyses.

## Results

During the average 4.72 and 4.68 years of follow-up, we identified 765 and 1316 incidents of depression and anxiety, respectively. Compared with healthy participants, those with depression were more likely to be female (65.4%), never smoking (84.7%), never drinking (88.0%), and demonstrated a higher incidence of hypertension, dyslipidemia, cancer, and stroke (Table 1). Those with anxiety tended to be older, female, less educated, nonsmokers, never drinking, lower income, and demonstrated a higher proportion of hypertension, diabetes, dyslipidemia, cancer, and stroke and consumed less seafood and meat. Exposure to aluminum in drinking water was higher in participants with depression than in healthy ones (Table 2). Exposure to manganese, iron, and aluminum in drinking water was higher among anxiety participants, whereas zinc exposure was lower than healthy participants. Individuals residing in areas with higher concentrations of heavy metals in tap water have lower levels of socioeconomic level, especially copper (Additional file 1: Tables S1-S8).

**Table 1:** Characteristics of study participants

Characteristics	Healthy people (*N* = 22,203)	Depression (*N* = 765)	Anxiety (*N* = 1316)	*P* value
Age, *n* (%)				< 0.001
Under 55	5949 (26.8%)	179 (23.4%)	210 (16.0%)	
55 ~ 65	8190 (36.9%)	294 (38.4%)	492 (37.4%)	
Over 65	8064 (36.3%)	292 (38.2%)	614 (46.7%)	
Sex, *n* (%)				< 0.001
Male	9335 (42.0%)	265 (34.6%)	448 (34.0%)	
Education, *n* (%)				< 0.001
Primary school and below	16,226 (73.1%)	575 (75.2%)	1040 (79.0%)	
Middle school and above	5977 (26.9%)	190 (24.8%)	276 (21.0%)	
BMI, *n* (%)				0.86
Underweight	859 (3.9%)	36 (4.7%)	48 (3.6%)	
Normal	14,005 (63.1%)	473 (61.8%)	832 (63.2%)	
Overweight	6630 (29.9%)	234 (30.6%)	395 (30.0%)	
Obesity	709 (3.2%)	22 (2.9%)	41 (3.1%)	
Smoking status, *n* (%)				< 0.001
Never	17,471 (78.7%)	648 (84.7%)	1090 (82.8%)	
Current	3905 (17.6%)	93 (12.2%)	182 (13.8%)	
Former	827 (3.7%)	24 (3.1%)	44 (3.3%)	
Alcohol drinking, *n* (%)				< 0.001
Never	18,129 (81.7%)	673 (88.0%)	1153 (87.6%)	
Current	3852 (17.3%)	86 (11.2%)	149 (11.3%)	
Former	222 (1.0%)	6 (0.8%)	14 (1.1%)	
Tea drinking, *n* (%)				0.04
Never	19,938 (89.8%)	701 (91.6%)	1213 (92.2%)	
Current	2162 (9.7%)	61 (8.0%)	98 (7.4%)	
Former	103 (0.5%)	3 (0.4%)	5 (0.4%)	
Annual income, *n* (%)				< 0.001
Under $4000	8895 (40.1%)	311 (40.7%)	681 (51.7%)	
Over $4000	13,308 (59.9%)	454 (59.3%)	635 (48.3%)	
Hypertension, *n* (%)	13,915 (62.7%)	566 (74.0%)	961 (73.0%)	< 0.001
Diabetes, *n* (%)	3806 (17.1%)	145 (19.0%)	265 (20.1%)	0.01
Dyslipidemia, *n* (%)	7200 (32.4%)	377 (49.3%)	528 (40.1%)	< 0.001
Cancer, *n* (%)	622 (2.8%)	33 (4.3%)	42 (3.2%)	0.04
Stroke, *n* (%)	810 (3.6%)	41 (5.4%)	52 (4.0%)	0.04
Vegetables, *n* (%)				< 0.001
≥ 6 days/week	11,287 (50.8%)	378 (49.4%)	738 (56.1%)	
4 ~ 5 days/week	10,285 (46.3%)	363 (47.5%)	532 (40.4%)	
≤ 3 days/week	631 (2.8%)	24 (3.1%)	46 (3.5%)	
Fruit, *n* (%)				0.01
≥ 4 times/week	4977 (22.4%)	205 (26.8%)	335 (25.5%)	
2 ~ 3 times/week	14,808 (66.7%)	474 (61.0%)	853 (64.8%)	
≤ 1 time/week	2418 (10.9%)	86 (11.2%)	128 (9.7%)	
Seafood, *n* (%)				< 0.001
≥ 4 times/week	5385 (24.3%)	163 (21.3%)	244 (18.5%)	
2 ~ 3 times/week	13,454 (60.6%)	467 (61.0%)	790 (60.0%)	
≤ 1 time/week	3364 (15.2%)	135 (17.6%)	282 (21.4%)	
Freshwater products, *n* (%)				< 0.001
≥ 4 times/week	4788 (21.6%)	128 (16.7%)	158 (12.0%)	
2 ~ 3 times/week	13,425 (60.5%)	470 (61.4%)	800 (60.8%)	
≤ 1 time/week	3990 (18.0%)	167 (21.8%)	358 (27.2%)	
Marine products, *n* (%)				< 0.001
≥ 4 times/week	7725 (34.8%)	229 (29.9%)	298 (22.6%)	
2 ~ 3 times/week	12,264 (55.2%)	443 (57.9%)	814 (61.9%)	
≤ 1 time/week	2214 (10.0%)	93 (12.2%)	204 (15.5%)	
Meat, *n* (%)				< 0.001
≥ 4 times/week	7700 (34.7%)	229 (29.9%)	329 (25.0%)	
2 ~ 3 times/week	12,224 (55.1%)	447 (58.4%)	751 (57.1%)	
≤ 1 time/week	2279 (10.3%)	89 (11.6%)	236 (17.9%)	
Red meat, *n* (%)				< 0.001
≥ 4 times/week	6924 (31.2%)	207 (27.1%)	303 (23.0%)	
2 ~ 3 times/week	12,264 (55.2%)	433 (56.6%)	733 (55.7%)	
≤ 1 time/week	3015 (13.6%)	125 (16.3%)	280 (21.3%)	
White meat, *n* (%)				< 0.001
≥ 4 times/week	3129 (14.1%)	93 (12.2%)	146 (11.1%)	
2 ~ 3 times/week	15,085 (67.9%)	512 (66.9%)	821 (62.4%)	
≤ 1 time/week	3989 (18.0%)	160 (20.9%)	349 (26.5%)	

Categorical variables are expressed as counts and percentage

*BMI* body mass index

**Table 2:** Exposures component comparison in drinking water

Exposures	Healthy people (*N* = 22,203)	Depression (*N* = 765)	Anxiety (*N* = 1316)	*P* value
Manganese (μg/day)	30.17 (15.00, 32.05)	30.17 (15.94, 32.05)	32.05 (30.17, 95.53)	< 0.001
Zinc (μg/day)	18.73 (7.92, 33.63)	18.73 (7.92, 33.63)	8.05 (5.03, 24.00)	< 0.001
Copper (μg/day)	20.11 (10.00, 20.11)	20.11 (10.00, 20.11)	20.11 (10.00, 20.11)	< 0.001
Iron (μg/day)	40.23 (32.50, 65.37)	40.23 (34.38, 65.37)	55.00 (40.23, 69.14)	< 0.001
Aluminum (μg/day)	27.75 (21.00, 40.98)	34.44 (23.63, 45.25)	34.44 (23.63, 42.24)	< 0.001
Cadmium (μg/day)	0.50 (0.29, 0.59)	0.50 (0.31, 0.62)	0.50 (0.32, 0.62)	< 0.001
Selenium (μg/day)	0.40 (0.20, 0.40)	0.40 (0.20, 0.40)	0.40 (0.20, 0.40)	< 0.001
Fluorine (mg/day)	0.21 (0.13, 0.25)	0.21 (0.13, 0.25)	0.20 (0.13, 0.25)	0.07

Continuous variables are described as the median (Q1, Q3)

**Fig. 2:**
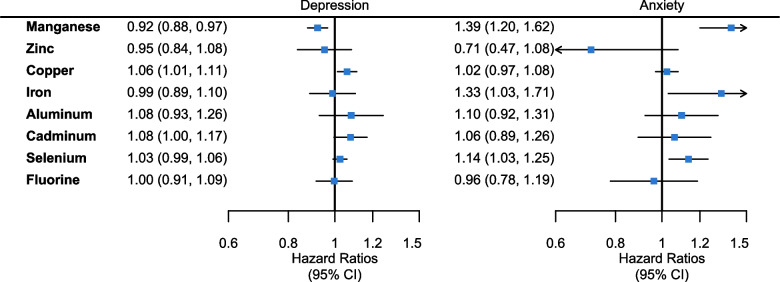
Prospective association between long-term exposure to metal and nonmetal elements and risk of depression and anxiety onset. Model adjusted for age; education; BMI; annual household income; current smoking status; current alcohol consumption status; current tea consumption; medical history of stroke, diabetes, hypertension, cancer, and dyslipidemia; current vegetable consumption; current fruit consumption; current seafood consumption; current freshwater product consumption; current marine product consumption; current meat consumption; current red meat consumption; and current white meat consumption

In the fully adjusted model, a 1 SD increase in manganese exposure correlated with an 8% decrease in depression incidence (HR 0.92, 95%CI 0.88 to 0.97) (Fig. 2), while a 1 SD increase in copper and cadmium exposure increased depression incidence by 6% (HR 1.06, 95%CI 1.01 to 1.11) and 8% (HR 1.08, 95%CI 1.00 to 1.17), respectively. There were no significant associations between depression and long-term exposure to zinc, iron, aluminum, selenium, and fluorine. Conversely, a 1 SD increase in manganese, iron, and selenium exposure increased the incidence of anxiety by 39% (HR 1.39, 95%CI 1.20 to 1.62), 33% (HR 1.33, 95%CI 1.03 to 1.71), and 14% (HR 1.14, 95%CI 1.03 to 1.25), respectively. Long-term exposure to zinc, copper, aluminum, cadmium, and fluorine demonstrated no significant correlation with anxiety.

**Fig. 3:**
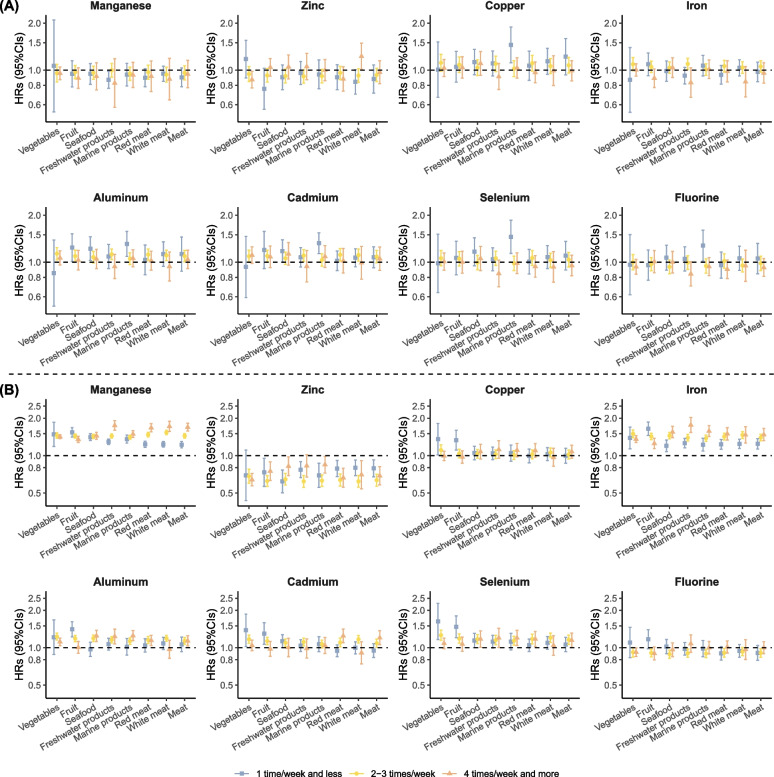
The moderating effect of food consumption on the prospective associations between metal and nonmetal elements and mental diseases. **A** Depression. **B** Anxiety. Model adjusted for age; education; BMI; annual household income; current smoking status; current alcohol consumption status; current tea consumption; medical history of stroke, diabetes, hypertension, cancer, and dyslipidemia; current vegetable consumption; current fruit consumption; current seafood consumption; current freshwater product consumption; current marine product consumption; current meat consumption; current red meat consumption; and current white meat consumption

The modification analyses suggested that no significant effect of diets on the relationships between manganese, copper, cadmium, and depression. The association between manganese, iron, and anxiety was higher in participants who ate fewer fruits, more marine products (seafood and freshwater), and meat (red meat, white meat). Additionally, long-term exposure to copper, selenium, and fluorine was associated with a higher risk of anxiety for participants who ate fewer green leafy vegetables and fewer fruits (Fig. 3).

Stratification analyses for the associations of exposure to metal and nonmetal elements in drinking water and metal diseases were conducted by age, income, education, smoking status, alcohol drinking status, tea drinking status, and BMI. A stronger association between exposure to cadmium in drinking water and depression was found in older, low-income, and less educated participants (Additional file 1: Table S9). For a 1 SD increase in long-term exposure to cadmium in drinking water, the incidence of depression increased by 23% (HR 1.23, 95% 1.06 to 1.43) in the participants over 65 years, 16% (HR 1.16, 95% 1.01 to 1.43) in the low-income participants, and 10% (HR 1.10, 95% 1.00 to 1.21) in the less educated participant. The associations between exposure to manganese, selenium, and anxiety were higher in younger participants and participants with relatively higher education levels (Additional file 1: Table S10). In contrast, a stronger association between exposure to iron in drinking water and anxiety was found in older and less educated participants. We found that the associations between manganese, iron, and anxiety were higher in low-income participants. The associations between manganese, iron, selenium, and anxiety were lower in participants who never smoked or drank.

Sensitivity analyses that excluded the events within the first years of follow-up showed similar results and the associations became more prominent. Most of the associations between exposure to metal and nonmetal elements in drinking water and depression still exist, while the association between manganese and depression was statistically insignificant (Additional file 1: Table S11). The E values of significant exposures were all more than 1.30. The E value is the minimum strength of association that the unmeasured confounders should have with both exposure and outcome in order to explain away the observed association, which means that the observed HRs were fully explained away by the unmeasured confounding effect when the unmeasured confounder and the exposure and the causal both have HRs of at least 1.30. To fully explain the association, the unmeasured confounding must be strongly associated with both the exposure and outcome (Additional file 1: Table S12). These results confirmed that our results are robust.

## Discussion

Our study found that long-term manganese exposure in drinking water reduces the incidence of depression, while copper and cadmium increase it. Additionally, exposure to manganese, iron, and selenium increased anxiety risk. We also found that diets had a significant modification effect on the relationship between long-term exposure to metal and nonmetal elements in drinking and the risk of anxiety. Considering the growing epidemic of depression and anxiety, our findings may shed light on the pathophysiology of depression and anxiety and tailor public health actions to improve the standard of drinking water and forming healthy eating habits for alleviating the disease burden of depression and anxiety. The public health policy should pay more attention to people with low SES to alleviate the disproportionate effect of metal and nonmetal elements on metal diseases.

The biological plausibility of the associations between metal elements and depression remains unclear. Previous studies reported a negative relationship between dietary manganese intake and depression [13, 32, 33]. Manganese is an essential component of manganese superoxide dismutase (MnSOD), which protects cells from oxidative stress [34], and deficiency intake of manganese may lead to depressive-like behaviors through the dopaminergic and serotonergic neurotransmission systems [35]. Our study suggests that copper and cadmium in drinking water might be risk factors for depression. Copper may affect the incidence of depression in many ways. Long-term exposure to copper in drinking water may promote the degradation of 5-hydroxytryptamine (5-HT) and decrease the 5-HT in vivo, affecting the activity and content of Cu/Zn superoxide dismutase and leading to neurological dysfunction [36]. Cadmium has been shown to be a risk factor for depression, with potential biological mechanisms involving dysregulation of the hypothalamic–pituitary–adrenal (HPA) axis, damage to the blood–brain barrier, and induction of neuron apoptosis [17, 37].

The association between metal and nonmetal elements and anxiety was an ongoing area of intense research interest. Animal studies have indicated that manganese administration can lead to anxiety-like behaviors and affect the dopaminergic and glutamatergic systems [38, 39]. The potential reason for the opposite associations of manganese with depression and anxiety is the difference in pathogenesis mechanisms, and manganese has different effects on the norepinephrine and dopamine neurotransmitters [40]. Previous cross-sectional studies showed that adults with iron deficiency anemia reported greater anxiety symptoms [41], which contradicts our findings, and a study from Bangladesh adults showed that long-term exposure to iron increased the risk for anxiety [42]. Iron overload has been confirmed with neurotransmitter homeostasis disruption, which may alter emotional behaviors and anxiety [43]. The potential explanations for this discrepancy may include the differences in study design and study participants. More studies with prospective design and nationally representative data are needed to further explore the association between iron and anxiety. Some studies have investigated the association between selenium and anxiety and found a protective association between selenium and anxiety. However, most of the studies were conducted in patients with comorbidities and anxiety was not the primary outcome [44, 45]. Our study suggests long-term zinc exposure from drinking water may decrease anxiety risk, although the association was not significant in the adjusted model. Preclinical studies have also indicated that zinc deficiency can influence anxiogenic-like behavior and cause hyperactivation of neurons, leading to mental disorders [12, 46].

We found that diets significantly modulate the relationships between metal and nonmetal elements and anxiety. The protective effect of green leafy vegetables and fruits may alleviate the risks of long-term exposure to metal and nonmetal elements in drinking water and anxiety. Numerous studies have shown that fruits and vegetables are significant protective factors for developing mental diseases [22]. Folate from fruits and vegetables plays an important role in producing neurotransmitters, including serotonin, dopamine, and epinephrine, which are important for mood regulation [47]. The protective effect of these factors may reduce the impact of exposure to metal and nonmetal elements in drinking water on mental disorders. A systematic analysis showed that red and processed meat significantly increased the risk of depression [48]. The potential explanations for the findings include the high intake of saturated fatty acids associated with low neuroplasticity and cognitive ability [49]. Additionally, the enrichment of metal and nonmetal elements in marine products further increases the level of exposure, influencing the effect of exposure to these elements in drinking water on anxiety [50]. In summary, increasing the intake of vegetables and fruits in the daily diet and consuming moderate amounts of meat and aquatic products has a potential impact on reducing the development of anxiety.

In the current study, we further suggest that the associations between exposure to cadmium in drinking water and the incidence of depression were more pronounced in older, low-income, and less educated participants. The potential explanations may include multiple pathways. Firstly, participants with low socioeconomic status (SES) may have poorer source water quality and more vivo exposure levels to metal and nonmetal elements [51]. Secondly, SES is a crucial determinant of mental disorders, with low SES associated with more psychosocial stress, negative emotions, and adverse events [52]. Thirdly, people with higher SES may have better social support and more health services utilization [53, 54]. The stratification analyses showed that the associations between selenium and anxiety were higher in high SES participants. The reason for the discrepancy of associations of cadmium and selenium with anxiety may be that people with high SES have a higher intake of selenium and a lower intake of cadmium in daily life, leading to the difference in serum concentrations of cadmium and selenium in people with different levels of SES [55, 56]. In contrast, exposure to iron and the risk of anxiety were more elevated in low SES participants. We found that the associations between manganese, iron, selenium, and anxiety were lower in participants who never smoked or drank. The potential explanation is that smoking and drinking have been proven as risk factors for anxiety [57], aggravating the hazards of metal and nonmetal elements on the risk of anxiety. However, more prospective and experimental studies are needed to explore the mechanisms of SES and behaviors on the relationships between metal and nonmetal elements and anxiety.

Our study results should be interpreted with several limitations. First, we used the measurement values from the nearest tap water pipeline from the participants’ residential addresses and the total daily drinking water intake of adults by sex to calculate the exposures. The total daily drinking water intake was calculated based on the parameters from regional levels, not the actual levels to which individuals are exposed. The exposure levels were aggregated in residential areas rather than linked to individual participants, which may limit the causality of the associations. Second, we assumed that the residents were all permanent residents and had no information about whether the drinking water was further filtered before drinking. Third, we could not obtain participants’ blood levels of metals and nonmetals due to data limitations. Future studies are needed to further explore the associations of serum concentrations of metals and nonmetals with mental diseases. In terms of strengths, this study is the first prospective study to examine the association between metal and nonmetal elements in drinking water and the risk of depression and anxiety. The hospital-based clinical diagnosis of depression and anxiety measurement alleviated the measurement bias caused by self-report or other ways.

## Conclusions

In conclusion, this study provides novel evidence that long-term exposure to metal and nonmetal elements in drinking water had significant associations with depression and anxiety. Eating more green leafy vegetables and fruits may alleviate the effect of metal and nonmetal elements in drinking water on mental disorders. Given the growing burden of mental disorders, our study sheds light on tailored public health policies for improving drinking water standards and keeping healthy diets to alleviate depression and anxiety impairment.

### Supplementary Information

**Additional file 1: Table S1.** Baseline characteristics of study participants stratified by the levels of Manganese in tap water. **Table S2.** Baseline characteristics of study participants stratified by the levels of Zinc in tap water. **Table S3.** Baseline characteristics of study participants stratified by the levels of Copper in tap water. **Table S4.** Baseline characteristics of study participants stratified by the levels of Iron in tap water. **Table S5.** Baseline characteristics of study participants stratified by the levels of Aluminum in tap water. **Table S6.** Baseline characteristics of study participants stratified by the levels of Cadmium in tap water. **Table S7.** Baseline characteristics of study participants stratified by the levels of Selenium in tap water. **Table S8.** Baseline characteristics of study participants stratified by the levels of Fluorine in tap water. **Table S9.** Subgroup analysis for the prospective association between metal, nonmetal elements exposure and onset of depression. **Table S10.** Subgroup analysis for the prospective association between metal, nonmetal elements exposure and onset of anxiety. **Table S11.** Sensitive analysis of association between long term exposure to metal and nonmetal elements and risk of depression and anxiety onset. **Table S12.** E-value for point estimates and the lower 95% confidence intervals of the Hazard. **Fig. S1.** Directed acyclic graph for water exposure and depression, anxiety.

## Data Availability

Please get in touch with the corresponding authors for more information.

## Abbreviations

5-HT: 5-Hydroxytryptamine
BMI: Body mass index
CI: Confidence intervals
DALYs: Disability-adjusted life years
HPA: Hypothalamic-pituitary-adrenal
HRs: Hazard ratios
MnSOD: Manganese superoxide dismutase
SD: Standard deviation
SES: Socioeconomic status
YHIS: Yinzhou Health Information System
