# Supplementary material for: Association between drinking water quality and mental health and the modifying role of diet: a prospective cohort study
Source: BMC Med. 2024 Feb 2;22:53. doi: 10.1186/s12916-024-03269-3 (PMC10835879; doi:10.1186/s12916-024-03269-3)
Supplement: Supplementary file 1 — Additional file 1: Table S1. Baseline characteristics of study participants stratified by the levels of Manganese in tap water. Table S2. Baseline characteristics of study participants stratified by the levels of Zinc in tap water. Table S3. Baseline characteristics of study participants stratified by the levels of Copper in tap water. Table S4. Baseline characteristics of study participants stratified by the levels of Iron in tap water. Table S5. Baseline characteristics of study participants stratified by the levels of Aluminum in tap water. Table S6. Baseline characteristics of study participants stratified by the levels of Cadmium in tap water. Table S7. Baseline characteristics of study participants stratified by the levels of Selenium in tap water. Table S8. Baseline characteristics of study participants stratified by the levels of Fluorine in tap water. Table S9. Subgroup analysis for the prospective association between metal, nonmetal elements exposure and onset of depression. Table S10. Subgroup analysis for the prospective association between metal, nonmetal elements exposure and onset of anxiety. Table S11. Sensitive analysis of association between long term exposure to metal and nonmetal elements and risk of depression and anxiety onset. Table S12. E-value for point estimates and the lower 95% confidence intervals of the Hazard. Fig. S1. Directed acyclic graph for water exposure and depression, anxiety. [file 12916_2024_3269_MOESM1_ESM.docx]

**Additional file 1**

**Supplementary Tables and Figures Legends**

Table S1. Baseline characteristics of study participants stratified by the levels of Manganese in tap water

Table S2. Baseline characteristics of study participants stratified by the levels of Zinc in tap water

Table S3. Baseline characteristics of study participants stratified by the levels of Copper in tap water

Table S4. Baseline characteristics of study participants stratified by the levels of Iron in tap water

Table S5. Baseline characteristics of study participants stratified by the levels of Aluminum in tap water

Table S6. Baseline characteristics of study participants stratified by the levels of Cadmium in tap water

Table S7. Baseline characteristics of study participants stratified by the levels of Selenium in tap water

Table S8. Baseline characteristics of study participants stratified by the levels of Fluorine in tap water

Table S9. Subgroup analysis for the prospective association between metal, nonmetal elements exposure and onset of depression

Table S10. Subgroup analysis for the prospective association between metal, nonmetal elements exposure and onset of anxiety

Table S11**.** Sensitive analysis of association between long term exposure to metal and nonmetal elements and risk of depression and anxiety onset

Table S12. E-value for point estimates and the lower 95% confidence intervals of the Hazard

Figure S1. Directed acyclic graph for water exposure and depression, anxiety

Table S1. Baseline characteristics of study participants stratified by the levels of Manganese in tap water.

| Characteristics | Manganese | | P Value |
| --- | --- | --- | --- |
|  | First Tertile  (The Lower) | Second Tertile  (The Higher) |  |
| Age, n (%) |  |  | <0.001 |
| Under 55 | 4119 (27.6%) | 2219 (23.7%) |  |
| 55~65 | 5526 (37.0%) | 3450 (36.8%) |  |
| Over 65 | 5274 (35.4%) | 3696 (39.5%) |  |
| Sex, n (%) |  |  | <0.001 |
| Male | 5937 (39.8%) | 4111 (43.9%) |  |
| Education, n (%) |  |  | 0.002 |
| Primary school and below | 11062 (74.1%) | 6779 (72.4%) |  |
| Middle school and above | 3857 (25.9%) | 2586 (27.6%) |  |
| BMI, n (%) |  |  | 0.93 |
| Underweight | 571 (3.8%) | 372 (4.0%) |  |
| Normal | 9400 (63.0%) | 5910 (63.1%) |  |
| Overweight | 4473 (30.0%) | 2786 (29.7%) |  |
| Obesity | 475 (3.2%) | 297 (3.2%) |  |
| Smoking status, n (%) |  |  | <0.001 |
| Never | 12026 (80.6%) | 7183 (76.7%) |  |
| Current | 2463 (16.5%) | 1717 (18.3%) |  |
| Former | 430 (2.9%) | 465 (5.0%) |  |
| Alcohol drinking, n (%) |  |  | <0.001 |
| Never | 12333 (82.7%) | 7622 (81.4%) |  |
| Current | 2467 (16.5%) | 1620 (17.3%) |  |
| Former | 119 (0.8%) | 123 (1.3%) |  |
| Tea drinking, n (%) |  |  | <0.001 |
| Never | 13431 (90.0%) | 8421 (89.9%) |  |
| Current | 1443 (9.7%) | 878 (9.4%) |  |
| Former | 45 (0.3%) | 66 (0.7%) |  |
| Annual income, n (%) |  |  | <0.001 |
| Under $4,000 | 5538 (37.1%) | 4349 (46.4%) |  |
| Over $4,000 | 9381 (62.9%) | 5016 (53.6%) |  |
| Hypertension, n (%) | 9222 (61.8%) | 6220 (66.4%) | <0.001 |
| Diabetes, n (%) | 2512 (16.8%) | 1704 (18.2%) | 0.007 |
| Dyslipidemia, n (%) | 4589 (30.8%) | 3516 (37.5%) | <0.001 |
| Cancer, n (%) | 436 (2.9%) | 261 (2.8%) | 0.54 |
| Stroke, n (%) | 574 (3.8%) | 329 (3.5%) | 0.18 |
| Vegetables, n (%) |  |  | <0.001 |
| ≥6 days/week | 6723 (45.1%) | 5680 (60.7%) |  |
| 4~5 days/week | 7728 (51.8%) | 3452 (36.9%) |  |
| ≤3 days/week | 468 (3.1%) | 233 (2.5%) |  |
| Fruit, n (%) |  |  | <0.001 |
| ≥4 times/week | 2623 (17.6%) | 2894 (30.9%) |  |
| 2~3 times/week | 10899 (73.1%) | 5236 (55.9%) |  |
| ≤1 time/week | 1397 (9.4%) | 1235 (13.2%) |  |
| Seafood, n (%) |  |  | <0.001 |
| ≥4 times/week | 3684 (24.7%) | 2108 (22.5%) |  |
| 2~3 times/week | 9623 (64.5%) | 5088 (54.3%) |  |
| ≤1 time/week | 1612 (10.8%) | 2169 (23.2%) |  |
| Freshwater products, n (%) |  |  | <0.001 |
| ≥4 times/week | 3743 (25.1%) | 1331 (14.2%) |  |
| 2~3 times/week | 9239 (61.9%) | 5456 (58.3%) |  |
| ≤1 time/week | 1937 (13.0%) | 2578 (27.5%) |  |
| Marine products, n (%) |  |  | <0.001 |
| ≥4 times/week | 5767 (38.7%) | 2485 (26.5%) |  |
| 2~3 times/week | 8058 (54.0%) | 5463 (58.3%) |  |
| ≤1 time/week | 1094 (7.3%) | 1417 (15.1%) |  |
| Meat, n (%) |  |  | <0.001 |
| ≥4 times/week | 5642 (37.8%) | 2616 (27.9%) |  |
| 2~3 times/week | 8038 (53.9%) | 5384 (57.5%) |  |
| ≤1 time/week | 1239 (8.3%) | 1365 (14.6%) |  |
| Red meat, n (%) |  |  | <0.001 |
| ≥4 times/week | 5203 (34.9%) | 2231 (23.8%) |  |
| 2~3 times/week | 8136 (54.5%) | 5294 (56.5%) |  |
| ≤1 time/week | 1580 (10.6%) | 1840 (19.6%) |  |
| White meat, n (%) |  |  | <0.001 |
| ≥4 times/week | 1988 (13.3%) | 1380 (14.7%) |  |
| 2~3 times/week | 10919 (73.2%) | 5499 (58.7%) |  |
| ≤1 time/week | 2012 (13.5%) | 2486 (26.5%) |  |

Table S2. Baseline characteristics of study participants stratified by the levels of Zinc in tap water.

| Characteristics | Zinc | | P Value |
| --- | --- | --- | --- |
|  | First Tertile  (The Lower) | Second Tertile  (The Higher) |  |
| Age, n (%) |  |  | <0.001 |
| Under 55 | 5209 (35.9%) | 1129 (11.5%) |  |
| 55~65 | 5245 (36.2%) | 3731 (38.2%) |  |
| Over 65 | 4052 (27.9%) | 4918 (50.3%) |  |
| Sex, n (%) |  |  | 0.025 |
| Male | 5918 (40.8%) | 4130 (42.2%) |  |
| Education, n (%) |  |  | <0.001 |
| Primary school and below | 9526 (65.7%) | 8315 (85.0%) |  |
| Middle school and above | 4980 (34.3%) | 1463 (15.0%) |  |
| BMI, n (%) |  |  | 0.89 |
| Underweight | 559 (3.9%) | 384 (3.9%) |  |
| Normal | 9143 (63.0%) | 6167 (63.1%) |  |
| Overweight | 4333 (29.9%) | 2926 (29.9%) |  |
| Obesity | 471 (3.2%) | 301 (3.1%) |  |
| Smoking status, n (%) |  |  | <0.001 |
| Never | 11300 (77.9%) | 7909 (80.9%) |  |
| Current | 2626 (18.1%) | 1554 (15.9%) |  |
| Former | 580 (4.0%) | 315 (3.2%) |  |
| Alcohol drinking, n (%) |  |  | <0.001 |
| Never | 12041 (83.0%) | 7914 (80.9%) |  |
| Current | 2306 (15.9%) | 1781 (18.2%) |  |
| Former | 159 (1.1%) | 83 (0.8%) |  |
| Tea drinking, n (%) |  |  | <0.001 |
| Never | 13196 (91.0%) | 8656 (88.5%) |  |
| Current | 1229 (8.5%) | 1092 (11.2%) |  |
| Former | 81 (0.6%) | 30 (0.3%) |  |
| Annual income, n (%) |  |  | <0.001 |
| Under $4,000 | 4970 (34.3%) | 4917 (50.3%) |  |
| Over $4,000 | 9536 (65.7%) | 4861 (49.7%) |  |
| Hypertension, n (%) | 8852 (61.0%) | 6590 (67.4%) | <0.001 |
| Diabetes, n (%) | 2263 (15.6%) | 1953 (20.0%) | <0.001 |
| Dyslipidemia, n (%) | 4747 (32.7%) | 3358 (34.3%) | 0.009 |
| Cancer, n (%) | 402 (2.8%) | 295 (3.0%) | 0.26 |
| Stroke, n (%) | 434 (3.0%) | 469 (4.8%) | <0.001 |
| Vegetables, n (%) |  |  | <0.001 |
| ≥6 days/week | 8307 (57.3%) | 4096 (41.9%) |  |
| 4~5 days/week | 5712 (39.4%) | 5468 (55.9%) |  |
| ≤3 days/week | 487 (3.4%) | 214 (2.2%) |  |
| Fruit, n (%) |  |  | <0.001 |
| ≥4 times/week | 4294 (29.6%) | 1223 (12.5%) |  |
| 2~3 times/week | 8727 (60.2%) | 7408 (75.8%) |  |
| ≤1 time/week | 1485 (10.2%) | 1147 (11.7%) |  |
| Seafood, n (%) |  |  | <0.001 |
| ≥4 times/week | 3400 (23.4%) | 2392 (24.5%) |  |
| 2~3 times/week | 8255 (56.9%) | 6456 (66.0%) |  |
| ≤1 time/week | 2851 (19.7%) | 930 (9.5%) |  |
| Freshwater products, n (%) |  |  | <0.001 |
| ≥4 times/week | 2947 (20.3%) | 2127 (21.8%) |  |
| 2~3 times/week | 8124 (56.0%) | 6571 (67.2%) |  |
| ≤1 time/week | 3435 (23.7%) | 1080 (11.0%) |  |
| Marine products, n (%) |  |  | <0.001 |
| ≥4 times/week | 4391 (30.3%) | 3861 (39.5%) |  |
| 2~3 times/week | 8200 (56.5%) | 5321 (54.4%) |  |
| ≤1 time/week | 1915 (13.2%) | 596 (6.1%) |  |
| Meat, n (%) |  |  | <0.001 |
| ≥4 times/week | 4564 (31.5%) | 3694 (37.8%) |  |
| 2~3 times/week | 8109 (55.9%) | 5313 (54.3%) |  |
| ≤1 time/week | 1833 (12.6%) | 771 (7.9%) |  |
| Red meat, n (%) |  |  | <0.001 |
| ≥4 times/week | 3996 (27.5%) | 3438 (35.2%) |  |
| 2~3 times/week | 8010 (55.2%) | 5420 (55.4%) |  |
| ≤1 time/week | 2500 (17.2%) | 920 (9.4%) |  |
| White meat, n (%) |  |  | <0.001 |
| ≥4 times/week | 2570 (17.7%) | 798 (8.2%) |  |
| 2~3 times/week | 8542 (58.9%) | 7876 (80.5%) |  |
| ≤1 time/week | 3394 (23.4%) | 1104 (11.3%) |  |

Table S3. Baseline characteristics of study participants stratified by the levels of Copper in tap water.

| Characteristics | Copper | | P Value |
| --- | --- | --- | --- |
|  | First Tertile  (The Lower) | Second Tertile  (The Higher) |  |
| Age, n (%) |  |  | <0.001 |
| Under 55 | 6242 (29.4%) | 96 (3.1%) |  |
| 55~65 | 7779 (36.7%) | 1197 (38.8%) |  |
| Over 65 | 7176 (33.9%) | 1794 (58.1%) |  |
| Sex, n (%) |  |  | <0.001 |
| Male | 8658 (40.8%) | 1390 (45.0%) |  |
| Education, n (%) |  |  | <0.001 |
| Primary school and below | 15167 (71.6%) | 2674 (86.6%) |  |
| Middle school and above | 6030 (28.4%) | 413 (13.4%) |  |
| BMI, n (%) |  |  | <0.001 |
| Underweight | 13539 (63.9%) | 1771 (57.4%) |  |
| Normal | 827 (3.9%) | 116 (3.8%) |  |
| Overweight | 6199 (29.2%) | 1060 (34.3%) |  |
| Obesity | 632 (3.0%) | 140 (4.5%) |  |
| Smoking status, n (%) |  |  | 0.012 |
| Never | 16705 (78.8%) | 2504 (81.1%) |  |
| Current | 3696 (17.4%) | 484 (15.7%) |  |
| Former | 796 (3.8%) | 99 (3.2%) |  |
| Alcohol drinking, n (%) |  |  | <0.001 |
| Never | 17521 (82.7%) | 2434 (78.8%) |  |
| Current | 3480 (16.4%) | 607 (19.7%) |  |
| Former | 196 (0.9%) | 46 (1.5%) |  |
| Tea drinking, n (%) |  |  | <0.001 |
| Never | 19159 (90.4%) | 2693 (87.2%) |  |
| Current | 1942 (9.2%) | 379 (12.3%) |  |
| Former | 96 (0.5%) | 15 (0.5%) |  |
| Annual income, n (%) |  |  | <0.001 |
| Under $4,000 | 7670 (36.2%) | 2217 (71.8%) |  |
| Over $4,000 | 13527 (63.8%) | 870 (28.2%) |  |
| Hypertension, n (%) | 13093 (61.8%) | 2349 (76.1%) | <0.001 |
| Diabetes, n (%) | 3495 (16.5%) | 721 (23.4%) | <0.001 |
| Dyslipidemia, n (%) | 6538 (30.8%) | 1567 (50.8%) | <0.001 |
| Cancer, n (%) | 579 (2.7%) | 118 (3.8%) | <0.001 |
| Stroke, n (%) | 680 (3.2%) | 223 (7.2%) | <0.001 |
| Vegetables, n (%) |  |  | <0.001 |
| ≥6 days/week | 11298 (53.3%) | 1105 (35.8%) |  |
| 4~5 days/week | 9318 (44.0%) | 1862 (60.3%) |  |
| ≤3 days/week | 581 (2.7%) | 120 (3.9%) |  |
| Fruit, n (%) |  |  | <0.001 |
| ≥4 times/week | 4942 (23.3%) | 575 (18.6%) |  |
| 2~3 times/week | 14084 (66.4%) | 2051 (66.4%) |  |
| ≤1 time/week | 2171 (10.2%) | 461 (14.9%) |  |
| Seafood, n (%) |  |  | <0.001 |
| ≥4 times/week | 5304 (25.0%) | 488 (15.8%) |  |
| 2~3 times/week | 12666 (59.8%) | 2045 (66.2%) |  |
| ≤1 time/week | 3227 (15.2%) | 554 (17.9%) |  |
| Freshwater products, n (%) |  |  | <0.001 |
| ≥4 times/week | 4529 (21.4%) | 545 (17.7%) |  |
| 2~3 times/week | 12597 (59.4%) | 2098 (68.0%) |  |
| ≤1 time/week | 4071 (19.2%) | 444 (14.4%) |  |
| Marine products, n (%) |  |  | <0.001 |
| ≥4 times/week | 7600 (35.9%) | 652 (21.1%) |  |
| 2~3 times/week | 11377 (53.7%) | 2144 (69.5%) |  |
| ≤1 time/week | 2220 (10.5%) | 291 (9.4%) |  |
| Meat, n (%) |  |  | <0.001 |
| ≥4 times/week | 7714 (36.4%) | 544 (17.6%) |  |
| 2~3 times/week | 11196 (52.8%) | 2226 (72.1%) |  |
| ≤1 time/week | 2287 (10.8%) | 317 (10.3%) |  |
| Red meat, n (%) |  |  | <0.001 |
| ≥4 times/week | 7027 (33.2%) | 407 (13.2%) |  |
| 2~3 times/week | 11151 (52.6%) | 2279 (73.8%) |  |
| ≤1 time/week | 3019 (14.2%) | 401 (13.0%) |  |
| White meat, n (%) |  |  | <0.001 |
| ≥4 times/week | 2868 (13.5%) | 500 (16.2%) |  |
| 2~3 times/week | 14233 (67.1%) | 2185 (70.8%) |  |
| ≤1 time/week | 4096 (19.3%) | 402 (13.0%) |  |

Table S4. Baseline characteristics of study participants stratified by the levels of Iron in tap water.

| Characteristics | Iron | | P Value |
| --- | --- | --- | --- |
|  | First Tertile  (The Lower) | Second Tertile  (The Higher) |  |
| Age, n (%) |  |  | 0.82 |
| Under 55 | 4025 (26.2%) | 2313 (25.9%) |  |
| 55~65 | 5658 (36.9%) | 3318 (37.1%) |  |
| Over 65 | 5661 (36.9%) | 3309 (37.0%) |  |
| Sex, n (%) |  |  | <0.001 |
| Male | 6136 (40.0%) | 3912 (43.8%) |  |
| Education, n (%) |  |  | <0.001 |
| Primary school and below | 11523 (75.1%) | 6318 (70.7%) |  |
| Middle school and above | 3821 (24.9%) | 2622 (29.3%) |  |
| BMI, n (%) |  |  | 0.41 |
| Underweight | 9655 (62.9%) | 5655 (63.3%) |  |
| Normal | 580 (3.8%) | 363 (4.1%) |  |
| Overweight | 4605 (30.0%) | 2654 (29.7%) |  |
| Obesity | 504 (3.3%) | 268 (3.0%) |  |
| Smoking status, n (%) |  |  | <0.001 |
| Never | 12384 (80.7%) | 6825 (76.3%) |  |
| Current | 2527 (16.5%) | 1653 (18.5%) |  |
| Former | 433 (2.8%) | 462 (5.2%) |  |
| Alcohol drinking, n (%) |  |  | <0.001 |
| Never | 12696 (82.7%) | 7259 (81.2%) |  |
| Current | 2526 (16.5%) | 1561 (17.5%) |  |
| Former | 122 (0.8%) | 120 (1.3%) |  |
| Tea drinking, n (%) |  |  | <0.001 |
| Never | 13828 (90.1%) | 8024 (89.8%) |  |
| Current | 1468 (9.6%) | 853 (9.5%) |  |
| Former | 48 (0.3%) | 63 (0.7%) |  |
| Annual income, n (%) |  |  | <0.001 |
| Under $4,000 | 5961 (38.8%) | 3926 (43.9%) |  |
| Over $4,000 | 9383 (61.2%) | 5014 (56.1%) |  |
| Hypertension, n (%) | 9533 (62.1%) | 5909 (66.1%) | <0.001 |
| Diabetes, n (%) | 2631 (17.1%) | 1585 (17.7%) | 0.25 |
| Dyslipidemia, n (%) | 4773 (31.1%) | 3332 (37.3%) | <0.001 |
| Cancer, n (%) | 453 (3.0%) | 244 (2.7%) | 0.32 |
| Stroke, n (%) | 602 (3.9%) | 301 (3.4%) | 0.027 |
| Vegetables, n (%) |  |  | <0.001 |
| ≥6 days/week | 7251 (47.3%) | 5152 (57.6%) |  |
| 4~5 days/week | 7637 (49.8%) | 3543 (39.6%) |  |
| ≤3 days/week | 456 (3.0%) | 245 (2.7%) |  |
| Fruit, n (%) |  |  | <0.001 |
| ≥4 times/week | 2769 (18.0%) | 2748 (30.7%) |  |
| 2~3 times/week | 11140 (72.6%) | 4995 (55.9%) |  |
| ≤1 time/week | 1435 (9.4%) | 1197 (13.4%) |  |
| Seafood, n (%) |  |  | <0.001 |
| ≥4 times/week | 3665 (23.9%) | 2127 (23.8%) |  |
| 2~3 times/week | 9776 (63.7%) | 4935 (55.2%) |  |
| ≤1 time/week | 1903 (12.4%) | 1878 (21.0%) |  |
| Freshwater products, n (%) |  |  | <0.001 |
| ≥4 times/week | 3727 (24.3%) | 1347 (15.1%) |  |
| 2~3 times/week | 9558 (62.3%) | 5137 (57.5%) |  |
| ≤1 time/week | 2059 (13.4%) | 2456 (27.5%) |  |
| Marine products, n (%) |  |  | <0.001 |
| ≥4 times/week | 5735 (37.4%) | 2517 (28.2%) |  |
| 2~3 times/week | 8386 (54.7%) | 5135 (57.4%) |  |
| ≤1 time/week | 1223 (8.0%) | 1288 (14.4%) |  |
| Meat, n (%) |  |  | <0.001 |
| ≥4 times/week | 5778 (37.7%) | 2480 (27.7%) |  |
| 2~3 times/week | 8251 (53.8%) | 5171 (57.8%) |  |
| ≤1 time/week | 1315 (8.6%) | 1289 (14.4%) |  |
| Red meat, n (%) |  |  | <0.001 |
| ≥4 times/week | 5354 (34.9%) | 2080 (23.3%) |  |
| 2~3 times/week | 8312 (54.2%) | 5118 (57.2%) |  |
| ≤1 time/week | 1678 (10.9%) | 1742 (19.5%) |  |
| White meat, n (%) |  |  | <0.001 |
| ≥4 times/week | 2058 (13.4%) | 1310 (14.7%) |  |
| 2~3 times/week | 11162 (72.7%) | 5256 (58.8%) |  |
| ≤1 time/week | 2124 (13.8%) | 2374 (26.6%) |  |

Table S5. Baseline characteristics of study participants stratified by the levels of Aluminum in tap water.

| Characteristics | Aluminum | | P Value |
| --- | --- | --- | --- |
|  | First Tertile  (The Lower) | Second Tertile  (The Higher) |  |
| Age, n (%) |  |  | <0.001 |
| Under 55 | 2937 (23.6%) | 3401 (28.7%) |  |
| 55~65 | 4526 (36.4%) | 4450 (37.5%) |  |
| Over 65 | 4959 (39.9%) | 4011 (33.8%) |  |
| Sex, n (%) |  |  | <0.001 |
| Male | 5280 (42.5%) | 4768 (40.2%) |  |
| Education, n (%) |  |  | 0.11 |
| Primary school and below | 9181 (73.9%) | 8660 (73.0%) |  |
| Middle school and above | 3241 (26.1%) | 3202 (27.0%) |  |
| BMI, n (%) |  |  | <0.001 |
| Underweight | 7995 (64.4%) | 7315 (61.7%) |  |
| Normal | 468 (3.8%) | 475 (4.0%) |  |
| Overweight | 3605 (29.0%) | 3654 (30.8%) |  |
| Obesity | 354 (2.8%) | 418 (3.5%) |  |
| Smoking status, n (%) |  |  | 0.056 |
| Never | 9901 (79.7%) | 9308 (78.5%) |  |
| Current | 2071 (16.7%) | 2109 (17.8%) |  |
| Former | 450 (3.6%) | 445 (3.8%) |  |
| Alcohol drinking, n (%) |  |  | <0.001 |
| Never | 10136 (81.6%) | 9819 (82.8%) |  |
| Current | 2186 (17.6%) | 1901 (16.0%) |  |
| Former | 100 (0.8%) | 142 (1.2%) |  |
| Tea drinking, n (%) |  |  | 0.001 |
| Never | 11093 (89.3%) | 10759 (90.7%) |  |
| Current | 1271 (10.2%) | 1050 (8.9%) |  |
| Former | 58 (0.5%) | 53 (0.4%) |  |
| Annual income, n (%) |  |  | <0.001 |
| Under $4,000 | 4783 (38.5%) | 5104 (43.0%) |  |
| Over $4,000 | 7639 (61.5%) | 6758 (57.0%) |  |
| Hypertension, n (%) | 8281 (66.7%) | 7161 (60.4%) | <0.001 |
| Diabetes, n (%) | 2422 (19.5%) | 1794 (15.1%) | <0.001 |
| Dyslipidemia, n (%) | 4506 (36.3%) | 3599 (30.3%) | <0.001 |
| Cancer, n (%) | 377 (3.0%) | 320 (2.7%) | 0.12 |
| Stroke, n (%) | 463 (3.7%) | 440 (3.7%) | 0.94 |
| Vegetables, n (%) |  |  | <0.001 |
| ≥6 days/week | 6261 (50.4%) | 6142 (51.8%) |  |
| 4~5 days/week | 5934 (47.8%) | 5246 (44.2%) |  |
| ≤3 days/week | 227 (1.8%) | 474 (4.0%) |  |
| Fruit, n (%) |  |  | <0.001 |
| ≥4 times/week | 2487 (20.0%) | 3030 (25.5%) |  |
| 2~3 times/week | 8492 (68.4%) | 7643 (64.4%) |  |
| ≤1 time/week | 1443 (11.6%) | 1189 (10.0%) |  |
| Seafood, n (%) |  |  | <0.001 |
| ≥4 times/week | 3301 (26.6%) | 2491 (21.0%) |  |
| 2~3 times/week | 7317 (58.9%) | 7394 (62.3%) |  |
| ≤1 time/week | 1804 (14.5%) | 1977 (16.7%) |  |
| Freshwater products, n (%) |  |  | <0.001 |
| ≥4 times/week | 2583 (20.8%) | 2491 (21.0%) |  |
| 2~3 times/week | 8053 (64.8%) | 6642 (56.0%) |  |
| ≤1 time/week | 1786 (14.4%) | 2729 (23.0%) |  |
| Marine products, n (%) |  |  | <0.001 |
| ≥4 times/week | 4986 (40.1%) | 3266 (27.5%) |  |
| 2~3 times/week | 6368 (51.3%) | 7153 (60.3%) |  |
| ≤1 time/week | 1068 (8.6%) | 1443 (12.2%) |  |
| Meat, n (%) |  |  | <0.001 |
| ≥4 times/week | 5319 (42.8%) | 2939 (24.8%) |  |
| 2~3 times/week | 6393 (51.5%) | 7029 (59.3%) |  |
| ≤1 time/week | 710 (5.7%) | 1894 (16.0%) |  |
| Red meat, n (%) |  |  | <0.001 |
| ≥4 times/week | 4867 (39.2%) | 2567 (21.6%) |  |
| 2~3 times/week | 6444 (51.9%) | 6986 (58.9%) |  |
| ≤1 time/week | 1111 (8.9%) | 2309 (19.5%) |  |
| White meat, n (%) |  |  | <0.001 |
| ≥4 times/week | 1326 (10.7%) | 2042 (17.2%) |  |
| 2~3 times/week | 9373 (75.5%) | 7045 (59.4%) |  |
| ≤1 time/week | 1723 (13.9%) | 2775 (23.4%) |  |

Table S6. Baseline characteristics of study participants stratified by the levels of Cadmium in tap water.

| Characteristics | Cadmium | | P Value |
| --- | --- | --- | --- |
|  | First Tertile  (The Lower) | Second Tertile  (The Higher) |  |
| Age, n (%) |  |  | <0.001 |
| Under 55 | 3188 (21.1%) | 3150 (34.3%) |  |
| 55~65 | 5697 (37.8%) | 3279 (35.7%) |  |
| Over 65 | 6206 (41.1%) | 2764 (30.1%) |  |
| Sex, n (%) |  |  | <0.001 |
| Male | 6443 (42.7%) | 3605 (39.2%) |  |
| Education, n (%) |  |  | <0.001 |
| Primary school and below | 11559 (76.6%) | 6282 (68.3%) |  |
| Middle school and above | 3532 (23.4%) | 2911 (31.7%) |  |
| BMI, n (%) |  |  | <0.001 |
| Underweight | 9687 (64.2%) | 5623 (61.2%) |  |
| Normal | 603 (4.0%) | 340 (3.7%) |  |
| Overweight | 4369 (29.0%) | 2890 (31.4%) |  |
| Obesity | 432 (2.9%) | 340 (3.7%) |  |
| Smoking status, n (%) |  |  | <0.001 |
| Never | 11789 (78.1%) | 7420 (80.7%) |  |
| Current | 2637 (17.5%) | 1543 (16.8%) |  |
| Former | 665 (4.4%) | 230 (2.5%) |  |
| Alcohol drinking, n (%) |  |  | <0.001 |
| Never | 12281 (81.4%) | 7674 (83.5%) |  |
| Current | 2647 (17.5%) | 1440 (15.7%) |  |
| Former | 163 (1.1%) | 79 (0.9%) |  |
| Tea drinking, n (%) |  |  | 0.003 |
| Never | 13512 (89.5%) | 8340 (90.7%) |  |
| Current | 1499 (9.9%) | 822 (8.9%) |  |
| Former | 80 (0.5%) | 31 (0.3%) |  |
| Annual income, n (%) |  |  | 0.002 |
| Under $4,000 | 6262 (41.5%) | 3625 (39.4%) |  |
| Over $4,000 | 8829 (58.5%) | 5568 (60.6%) |  |
| Hypertension, n (%) | 9827 (65.1%) | 5615 (61.1%) | <0.001 |
| Diabetes, n (%) | 2765 (18.3%) | 1451 (15.8%) | <0.001 |
| Dyslipidemia, n (%) | 4980 (33.0%) | 3125 (34.0%) | 0.11 |
| Cancer, n (%) | 428 (2.8%) | 269 (2.9%) | 0.68 |
| Stroke, n (%) | 538 (3.6%) | 365 (4.0%) | 0.11 |
| Vegetables, n (%) |  |  | <0.001 |
| ≥6 days/week | 7994 (53.0%) | 4409 (48.0%) |  |
| 4~5 days/week | 6795 (45.0%) | 4385 (47.7%) |  |
| ≤3 days/week | 302 (2.0%) | 399 (4.3%) |  |
| Fruit, n (%) |  |  | <0.001 |
| ≥4 times/week | 3228 (21.4%) | 2289 (24.9%) |  |
| 2~3 times/week | 10033 (66.5%) | 6102 (66.4%) |  |
| ≤1 time/week | 1830 (12.1%) | 802 (8.7%) |  |
| Seafood, n (%) |  |  | <0.001 |
| ≥4 times/week | 3918 (26.0%) | 1874 (20.4%) |  |
| 2~3 times/week | 9105 (60.3%) | 5606 (61.0%) |  |
| ≤1 time/week | 2068 (13.7%) | 1713 (18.6%) |  |
| Freshwater products, n (%) |  |  | <0.001 |
| ≥4 times/week | 2913 (19.3%) | 2161 (23.5%) |  |
| 2~3 times/week | 9357 (62.0%) | 5338 (58.1%) |  |
| ≤1 time/week | 2821 (18.7%) | 1694 (18.4%) |  |
| Marine products, n (%) |  |  | <0.001 |
| ≥4 times/week | 5642 (37.4%) | 2610 (28.4%) |  |
| 2~3 times/week | 7997 (53.0%) | 5524 (60.1%) |  |
| ≤1 time/week | 1452 (9.6%) | 1059 (11.5%) |  |
| Meat, n (%) |  |  | <0.001 |
| ≥4 times/week | 5540 (36.7%) | 2718 (29.6%) |  |
| 2~3 times/week | 7987 (52.9%) | 5435 (59.1%) |  |
| ≤1 time/week | 1564 (10.4%) | 1040 (11.3%) |  |
| Red meat, n (%) |  |  | <0.001 |
| ≥4 times/week | 5051 (33.5%) | 2383 (25.9%) |  |
| 2~3 times/week | 7991 (53.0%) | 5439 (59.2%) |  |
| ≤1 time/week | 2049 (13.6%) | 1371 (14.9%) |  |
| White meat, n (%) |  |  | <0.001 |
| ≥4 times/week | 1526 (10.1%) | 1842 (20.0%) |  |
| 2~3 times/week | 10614 (70.3%) | 5804 (63.1%) |  |
| ≤1 time/week | 2951 (19.6%) | 1547 (16.8%) |  |

Table S7. Baseline characteristics of study participants stratified by the levels of Selenium in tap water.

| Characteristics | Selenium | | P Value |
| --- | --- | --- | --- |
|  | First Tertile  (The Lower) | Second Tertile  (The Higher) |  |
| Age, n (%) |  |  | <0.001 |
| Under 55 | 5628 (24.2%) | 710 (69.8%) |  |
| 55~65 | 8670 (37.3%) | 306 (30.1%) |  |
| Over 65 | 8969 (38.5%) | 1 (0.1%) |  |
| Sex, n (%) |  |  | <0.001 |
| Male | 9680 (41.6%) | 368 (36.2%) |  |
| Education, n (%) |  |  | <0.001 |
| Primary school and below | 17481 (75.1%) | 360 (35.4%) |  |
| Middle school and above | 5786 (24.9%) | 657 (64.6%) |  |
| BMI, n (%) |  |  | 0.47 |
| Underweight | 14677 (63.1%) | 633 (62.2%) |  |
| Normal | 909 (3.9%) | 34 (3.3%) |  |
| Overweight | 6948 (29.9%) | 311 (30.6%) |  |
| Obesity | 733 (3.2%) | 39 (3.8%) |  |
| Smoking status, n (%) |  |  | <0.001 |
| Never | 18434 (79.2%) | 775 (76.2%) |  |
| Current | 3960 (17.0%) | 220 (21.6%) |  |
| Former | 873 (3.8%) | 22 (2.2%) |  |
| Alcohol drinking, n (%) |  |  | <0.001 |
| Never | 19074 (82.0%) | 881 (86.6%) |  |
| Current | 3954 (17.0%) | 133 (13.1%) |  |
| Former | 239 (1.0%) | 3 (0.3%) |  |
| Tea drinking, n (%) |  |  | <0.001 |
| Never | 21004 (90.3%) | 848 (83.4%) |  |
| Current | 2153 (9.3%) | 168 (16.5%) |  |
| Former | 110 (0.5%) | 1 (0.1%) |  |
| Annual income, n (%) |  |  | <0.001 |
| Under $4,000 | 9824 (42.2%) | 63 (6.2%) |  |
| Over $4,000 | 13443 (57.8%) | 954 (93.8%) |  |
| Hypertension, n (%) | 15052 (64.7%) | 390 (38.3%) | <0.001 |
| Diabetes, n (%) | 4127 (17.7%) | 89 (8.8%) | <0.001 |
| Dyslipidemia, n (%) | 7986 (34.3%) | 119 (11.7%) | <0.001 |
| Cancer, n (%) | 677 (2.9%) | 20 (2.0%) | 0.078 |
| Stroke, n (%) | 891 (3.8%) | 12 (1.2%) | <0.001 |
| Vegetables, n (%) |  |  | <0.001 |
| ≥6 days/week | 12295 (52.8%) | 108 (10.6%) |  |
| 4~5 days/week | 10274 (44.2%) | 906 (89.1%) |  |
| ≤3 days/week | 698 (3.0%) | 3 (0.3%) |  |
| Fruit, n (%) |  |  | <0.001 |
| ≥4 times/week | 5482 (23.6%) | 35 (3.4%) |  |
| 2~3 times/week | 15163 (65.2%) | 972 (95.6%) |  |
| ≤1 time/week | 2622 (11.3%) | 10 (1.0%) |  |
| Seafood, n (%) |  |  | <0.001 |
| ≥4 times/week | 5766 (24.8%) | 26 (2.6%) |  |
| 2~3 times/week | 13809 (59.4%) | 902 (88.7%) |  |
| ≤1 time/week | 3692 (15.9%) | 89 (8.8%) |  |
| Freshwater products, n (%) |  |  | <0.001 |
| ≥4 times/week | 5063 (21.8%) | 11 (1.1%) |  |
| 2~3 times/week | 13887 (59.7%) | 808 (79.4%) |  |
| ≤1 time/week | 4317 (18.6%) | 198 (19.5%) |  |
| Marine products, n (%) |  |  | <0.001 |
| ≥4 times/week | 8222 (35.3%) | 30 (2.9%) |  |
| 2~3 times/week | 12610 (54.2%) | 911 (89.6%) |  |
| ≤1 time/week | 2435 (10.5%) | 76 (7.5%) |  |
| Meat, n (%) |  |  | <0.001 |
| ≥4 times/week | 8242 (35.4%) | 16 (1.6%) |  |
| 2~3 times/week | 12638 (54.3%) | 784 (77.1%) |  |
| ≤1 time/week | 2387 (10.3%) | 217 (21.3%) |  |
| Red meat, n (%) |  |  | <0.001 |
| ≥4 times/week | 7420 (31.9%) | 14 (1.4%) |  |
| 2~3 times/week | 12735 (54.7%) | 695 (68.3%) |  |
| ≤1 time/week | 3112 (13.4%) | 308 (30.3%) |  |
| White meat, n (%) |  |  | <0.001 |
| ≥4 times/week | 3358 (14.4%) | 10 (1.0%) |  |
| 2~3 times/week | 15654 (67.3%) | 764 (75.1%) |  |
| ≤1 time/week | 4255 (18.3%) | 243 (23.9%) |  |

Table S8. Baseline characteristics of study participants stratified by the levels of Fluorine in tap water.

| Characteristics | Fluorine | | P Value |
| --- | --- | --- | --- |
|  | First Tertile  (The Lower) | Second Tertile  (The Higher) |  |
| Age, n (%) |  |  | <0.001 |
| Under 55 | 4804 (38.2%) | 1534 (13.1%) |  |
| 55~65 | 4465 (35.5%) | 4511 (38.6%) |  |
| Over 65 | 3318 (26.4%) | 5652 (48.3%) |  |
| Sex, n (%) |  |  | 0.004 |
| Male | 5099 (40.5%) | 4949 (42.3%) |  |
| Education, n (%) |  |  | <0.001 |
| Primary school and below | 8148 (64.7%) | 9693 (82.9%) |  |
| Middle school and above | 4439 (35.3%) | 2004 (17.1%) |  |
| BMI, n (%) |  |  | 0.26 |
| Underweight | 7966 (63.3%) | 7344 (62.8%) |  |
| Normal | 461 (3.7%) | 482 (4.1%) |  |
| Overweight | 3751 (29.8%) | 3508 (30.0%) |  |
| Obesity | 409 (3.2%) | 363 (3.1%) |  |
| Smoking status, n (%) |  |  | 0.023 |
| Never | 9880 (78.5%) | 9329 (79.8%) |  |
| Current | 2212 (17.6%) | 1968 (16.8%) |  |
| Former | 495 (3.9%) | 400 (3.4%) |  |
| Alcohol drinking, n (%) |  |  | <0.001 |
| Never | 10473 (83.2%) | 9482 (81.1%) |  |
| Current | 1977 (15.7%) | 2110 (18.0%) |  |
| Former | 137 (1.1%) | 105 (0.9%) |  |
| Tea drinking, n (%) |  |  | <0.001 |
| Never | 11495 (91.3%) | 10357 (88.5%) |  |
| Current | 1019 (8.1%) | 1302 (11.1%) |  |
| Former | 73 (0.6%) | 38 (0.3%) |  |
| Annual income, n (%) |  |  | <0.001 |
| Under $4,000 | 4070 (32.3%) | 5817 (49.7%) |  |
| Over $4,000 | 8517 (67.7%) | 5880 (50.3%) |  |
| Hypertension, n (%) | 7818 (62.1%) | 7624 (65.2%) | <0.001 |
| Diabetes, n (%) | 2006 (15.9%) | 2210 (18.9%) | <0.001 |
| Dyslipidemia, n (%) | 4341 (34.5%) | 3764 (32.2%) | <0.001 |
| Cancer, n (%) | 351 (2.8%) | 346 (3.0%) | 0.43 |
| Stroke, n (%) | 385 (3.1%) | 518 (4.4%) | <0.001 |
| Vegetables, n (%) |  |  | <0.001 |
| ≥6 days/week | 7245 (57.6%) | 5158 (44.1%) |  |
| 4~5 days/week | 4841 (38.5%) | 6339 (54.2%) |  |
| ≤3 days/week | 501 (4.0%) | 200 (1.7%) |  |
| Fruit, n (%) |  |  | <0.001 |
| ≥4 times/week | 4089 (32.5%) | 1428 (12.2%) |  |
| 2~3 times/week | 7304 (58.0%) | 8831 (75.5%) |  |
| ≤1 time/week | 1194 (9.5%) | 1438 (12.3%) |  |
| Seafood, n (%) |  |  | <0.001 |
| ≥4 times/week | 3215 (25.5%) | 2577 (22.0%) |  |
| 2~3 times/week | 6825 (54.2%) | 7886 (67.4%) |  |
| ≤1 time/week | 2547 (20.2%) | 1234 (10.5%) |  |
| Freshwater products, n (%) |  |  | <0.001 |
| ≥4 times/week | 2704 (21.5%) | 2370 (20.3%) |  |
| 2~3 times/week | 6822 (54.2%) | 7873 (67.3%) |  |
| ≤1 time/week | 3061 (24.3%) | 1454 (12.4%) |  |
| Marine products, n (%) |  |  | <0.001 |
| ≥4 times/week | 4145 (32.9%) | 4107 (35.1%) |  |
| 2~3 times/week | 6817 (54.2%) | 6704 (57.3%) |  |
| ≤1 time/week | 1625 (12.9%) | 886 (7.6%) |  |
| Meat, n (%) |  |  | <0.001 |
| ≥4 times/week | 4287 (34.1%) | 3971 (33.9%) |  |
| 2~3 times/week | 6890 (54.7%) | 6532 (55.8%) |  |
| ≤1 time/week | 1410 (11.2%) | 1194 (10.2%) |  |
| Red meat, n (%) |  |  | <0.001 |
| ≥4 times/week | 3729 (29.6%) | 3705 (31.7%) |  |
| 2~3 times/week | 6892 (54.8%) | 6538 (55.9%) |  |
| ≤1 time/week | 1966 (15.6%) | 1454 (12.4%) |  |
| White meat, n (%) |  |  | <0.001 |
| ≥4 times/week | 2378 (18.9%) | 990 (8.5%) |  |
| 2~3 times/week | 7176 (57.0%) | 9242 (79.0%) |  |
| ≤1 time/week | 3033 (24.1%) | 1465 (12.5%) |  |

Table S9. Subgroup analysis for the prospective association between metal, nonmetal elements exposure and onset of depression

|  | Manganese | Zinc | Copper | Iron | Aluminum | Cadmium | Selenium | Fluorine |
| --- | --- | --- | --- | --- | --- | --- | --- | --- |
| Age |  |  |  |  |  |  |  |  |
| Under 55 | 0.88 (0.35, 2.22) | 1.10 (0.97, 1.24) | 1.03 (0.86, 1.24) | 0.91 (0.76, 1.09) | 0.91 (0.77, 1.06) | 0.94 (0.82, 1.08) | 0.95 (0.82, 1.10) | 1.04 (0.87, 1.25) |
| 55~65 | 0.93 (0.83, 1.05) | 0.86 (0.76, 0.98) | 1.07 (0.93, 1.24) | 1.02 (0.92, 1.13) | 1.11 (0.99, 1.24) | 1.10 (0.99, 1.24) | 1.09 (0.95, 1.25) | 1.02 (0.89, 1.16) |
| Over 65 | 0.91 (0.82, 1.02) | 0.95 (0.84, 1.08) | 1.06 (0.93, 1.21) | 0.99 (0.89, 1.09) | **1.18 (1.06, 1.32)** | **1.23 (1.06, 1.43)** | 1.03 (0.89, 1.18) | 0.96 (0.86, 1.08) |
| Income |  |  |  |  |  |  |  |  |
| Under $4,000 | 0.94 (0.85, 1.03) | 0.93 (0.82, 1.04) | 1.06 (0.92, 1.21) | 0.99 (0.90, 1.08) | 1.09 (0.98, 1.21) | **1.16 (1.01, 1.34)** | 1.05 (0.91, 1.21) | 0.99 (0.88, 1.12) |
| Over $4,000 | 0.89 (0.77, 1.03) | 0.98 (0.88, 1.08) | 1.07 (0.95, 1.20) | 1.00 (0.90, 1.10) | 1.08 (0.97, 1.19) | 1.04 (0.95, 1.14) | 1.02 (0.91, 1.13) | 1.00 (0.90, 1.12) |
| Education |  |  |  |  |  |  |  |  |
| Primary and below | **0.92 (0.84, 1.00)** | 0.93 (0.85, 1.01) | 1.04 (0.93, 1.16) | 0.98 (0.90, 1.06) | **1.10 (1.01, 1.20)** | **1.10 (1.00, 1.21)** | 1.02 (0.91, 1.14) | 0.97 (0.88, 1.07) |
| Middle and above | 1.04 (0.79, 1.38) | 1.07 (0.91, 1.25) | 1.12 (0.95, 1.31) | 1.06 (0.89, 1.25) | 1.03 (0.90, 1.18) | 1.04 (0.92, 1.17) | 1.04 (0.91, 1.20) | 1.10 (0.94, 1.29) |
| Smoking status |  |  |  |  |  |  |  |  |
| Never | **0.92 (0.84, 1.00)** | 0.97 (0.89, 1.05) | 1.06 (0.96, 1.17) | 0.98 (0.91, 1.05) | **1.09 (1.00, 1.17)** | **1.09 (1.00, 1.18)** | 1.03 (0.94, 1.13) | 1.00 (0.91, 1.09) |
| Current | 1.08 (0.84, 1.40) | 0.71 (0.48, 1.07) | 1.25 (0.66, 2.35) | 1.34 (0.93, 1.93) | 0.99 (0.68, 1.46) | 0.85 (0.57, 1.26) | 0.69 (0.22, 2.12) | 1.08 (0.59, 2.00) |
| Former | 0.70 (0.39, 1.24) | 0.57 (0.23, 1.39) | 0.53 (0.02, 12.83) | 1.18 (0.76, 1.82) | 0.96 (0.44, 2.07) | 1.01 (0.47, 2.17) | 0.00 (0.00, 0.00) | 0.99 (0.42, 2.33) |
| Alcohol drinking |  |  |  |  |  |  |  |  |
| Never | 0.92 (0.84, 1.00) | 0.96 (0.88, 1.04) | 1.06 (0.96, 1.18) | 0.99 (0.91, 1.06) | **1.08 (1.00, 1.17)** | 1.08 (0.99, 1.17) | 1.03 (0.94, 1.13) | 1.00 (0.91, 1.09) |
| Current | 1.06 (0.80, 1.40) | 0.80 (0.51, 1.26) | 1.05 (0.67, 1.64) | 1.13 (0.83, 1.55) | 1.20 (0.91, 1.58) | 1.10 (0.82, 1.46) | 0.96 (0.57, 1.62) | 0.96 (0.56, 1.62) |
| Former | **0.01 (0.00, 0.13)** | 0.85 (0.41, 1.76) | 0.16 (0.00, 18.24) | 0.61 (0.26, 1.45) | 0.12 (0.01, 1.02) | 0.47 (0.13, 1.63) | 0.00 (0.00, 0.00) | 0.55 (0.18, 1.67) |
| Tea drinking |  |  |  |  |  |  |  |  |
| Never | **0.92 (0.84, 1.00)** | 0.96 (0.88, 1.04) | 1.06 (0.95, 1.17) | 0.98 (0.91, 1.06) | **1.08 (1.00, 1.17)** | 1.07 (0.99, 1.16) | 1.02 (0.93, 1.12) | 0.99 (0.91, 1.09) |
| Current | 1.03 (0.77, 1.39) | 0.69 (0.44, 1.08) | 1.19 (0.81, 1.75) | 1.23 (0.96, 1.57) | 1.12 (0.77, 1.63) | 1.18 (0.83, 1.68) | 1.21 (0.83, 1.77) | 1.09 (0.71, 1.66) |
| Former | 0.55 (0.24, 1.24) | 2.39 (1.16, 4.95) | 2.41 (0.71, 8.26) | 0.93 (0.35, 2.49) | 2.76 (1.00, 7.57) | 4.74 (0.80, 27.98) | 2.13 (0.58, 7.82) | 2.22 (0.75, 6.61) |
| BMI |  |  |  |  |  |  |  |  |
| Normal | 0.95 (0.86, 1.05) | 0.89 (0.80, 1.00) | 1.04 (0.92, 1.17) | 1.00 (0.91, 1.09) | 1.06 (0.97, 1.17) | 1.06 (0.96, 1.17) | 1.02 (0.91, 1.14) | 0.98 (0.88, 1.09) |
| Underweight | **0.63 (0.43, 0.92)** | 0.71 (0.47, 1.08) | 1.02 (0.73, 1.43) | 0.92 (0.73, 1.16) | 1.27 (0.90, 1.78) | 1.07 (0.77, 1.50) | 1.13 (0.75, 1.71) | 0.96 (0.69, 1.34) |
| Overweight | 0.92 (0.80, 1.05) | 1.06 (0.95, 1.20) | 1.11 (0.96, 1.30) | 0.98 (0.87, 1.11) | 1.09 (0.97, 1.22) | 1.11 (0.98, 1.25) | 1.04 (0.90, 1.19) | 1.05 (0.91, 1.21) |
| Obesity | 0.34 (0.15, 0.78) | 1.07 (0.76, 1.49) | 1.08 (0.65, 1.78) | 0.98 (0.74, 1.31) | 1.05 (0.71, 1.55) | 1.04 (0.71, 1.53) | 0.94 (0.62, 1.43) | 0.95 (0.60, 1.52) |

Model adjusted for age, education, BMI, annual household income, current smoking status, current alcohol consumption status, current tea consumption, medical history of stroke, diabetes, hypertension, cancer, dyslipidemia, current vegetable consumption, current fruit consumption, current seafood consumption, current freshwater product consumption, current marine product consumption, current meat consumption, current red meat consumption, and current white meat consumption.

Table S10. Subgroup analysis for the prospective association between metal, nonmetal elements exposure and onset of anxiety

|  | Manganese | Zinc | Copper | Iron | Aluminum | Cadmium | Selenium | Fluorine |
| --- | --- | --- | --- | --- | --- | --- | --- | --- |
| Age |  |  |  |  |  |  |  |  |
| Under 55 | **2.18 (1.22, 3.91)** | 0.94 (0.80, 1.12) | 1.19 (1.01, 1.40) | 0.96 (0.80, 1.14) | **1.13 (1.01, 1.26)** | **1.20 (1.10, 1.31)** | **1.24 (1.09, 1.40)** | 1.09 (0.93, 1.27) |
| 55~70 | **1.35 (1.27, 1.42)** | 0.82 (0.72, 0.92) | 1.03 (0.93, 1.14) | **1.32 (1.22, 1.44)** | **1.10 (1.01, 1.19)** | 1.05 (0.95, 1.15) | **1.14 (1.02, 1.27)** | 0.93 (0.84, 1.03) |
| Over 70 | **1.43 (1.37, 1.49)** | **0.58 (0.52, 0.65)** | 0.98 (0.90, 1.07) | **1.38 (1.29, 1.47)** | 1.08 (0.99, 1.18) | 0.96 (0.86, 1.06) | 1.08 (0.98, 1.20) | 0.95 (0.87, 1.04) |
| Income |  |  |  |  |  |  |  |  |
| Under $4,000 | **1.44 (1.38, 1.50)** | **0.60 (0.54, 0.67)** | 0.97 (0.89, 1.06) | **1.40 (1.31, 1.49)** | **1.09 (1.01, 1.17)** | 0.96 (0.87, 1.07) | 1.10 (0.99, 1.22) | 0.95 (0.87, 1.03) |
| Over $4,000 | **1.30 (1.22, 1.38)** | **0.89 (0.80, 1.00)** | **1.09 (1.00, 1.20)** | **1.20 (1.10, 1.31)** | **1.11 (1.03, 1.20)** | **1.12 (1.05, 1.20)** | **1.17 (1.07, 1.27)** | 0.99 (0.91, 1.08) |
| Education |  |  |  |  |  |  |  |  |
| Primary and below | **1.39 (1.34, 1.44)** | **0.71 (0.65, 0.78)** | 1.00 (0.92, 1.08) | 1.35 (1.27, 1.42) | 1.09 (1.02, 1.16) | 0.99 (0.92, 1.08) | 1.09 (1.00, 1.18) | 0.93 (0.87, 1.00) |
| Middle and above | **1.59 (1.41, 1.79)** | **0.73 (0.58, 0.92)** | **1.14 (1.00, 1.29)** | **1.20 (1.04, 1.39)** | **1.13 (1.02, 1.24)** | **1.19 (1.09, 1.30)** | **1.25 (1.12, 1.40)** | 1.11 (0.98, 1.27) |
| Smoking status |  |  |  |  |  |  |  |  |
| Never | **1.38 (1.33, 1.43)** | **0.73 (0.67, 0.80)** | 1.04 (0.96, 1.12) | **1.31 (1.24, 1.39)** | **1.09 (1.02, 1.15)** | 1.05 (0.99, 1.13) | **1.12 (1.04, 1.21)** | 0.97 (0.91, 1.04) |
| Current | **1.84 (1.61, 2.11)** | **0.47 (0.32, 0.70)** | 0.11 (0.00, 7.05) | **1.66 (1.31, 2.09)** | **1.36 (1.09, 1.70)** | 1.30 (0.97, 1.75) | **1.54 (1.19, 2.00)** | 0.68 (0.42, 1.11) |
| Former | **1.82 (1.46, 2.26)** | **0.45 (0.20, 0.99)** | 1.06 (0.13, 8.73) | **2.04 (1.50, 2.78)** | **1.90 (1.01, 3.55)** | 0.68 (0.14, 3.25) | 2.02 (0.93, 4.39) | 0.82 (0.19, 3.53) |
| Alcohol drinking |  |  |  |  |  |  |  |  |
| Never | **1.38 (1.33, 1.43)** | **0.72 (0.66, 0.78)** | 1.03 (0.96, 1.10) | **1.32 (1.25, 1.39)** | **1.08 (1.02, 1.15)** | 1.06 (0.99, 1.13) | **1.13 (1.04, 1.22)** | 0.97 (0.90, 1.04) |
| Current | **1.84 (1.61, 2.11)** | 0.74 (0.51, 1.08) | 0.93 (0.60, 1.42) | **1.60 (1.32, 1.93)** | **1.51 (1.26, 1.82)** | 1.21 (0.93, 1.57) | **1.35 (1.04, 1.76)** | 0.73 (0.42, 1.28) |
| Former | **1.86 (1.37, 2.54)** | **0.03 (0.00, 0.38)** | 1.17 (0.53, 2.61) | **1.98 (1.45, 2.69)** | 0.95 (0.32, 2.84) | 0.58 (0.13, 2.67) | 1.47 (0.69, 3.12) | 1.35 (0.73, 2.52) |
| Tea drinking |  |  |  |  |  |  |  |  |
| Never | **1.39 (1.34, 1.44)** | **0.72 (0.66, 0.79)** | 1.03 (0.96, 1.11) | **1.33 (1.26, 1.41)** | **1.09 (1.03, 1.16)** | 1.05 (0.98, 1.12) | **1.13 (1.04, 1.22)** | 0.96 (0.90, 1.03) |
| Current | **1.52 (1.30, 1.77)** | **0.52 (0.31, 0.87)** | 0.97 (0.67, 1.40) | **1.34 (1.07, 1.68)** | **1.32 (1.06, 1.65)** | **1.31 (1.05, 1.65)** | **1.39 (1.09, 1.77)** | 1.02 (0.71, 1.47) |
| Former | **1.18 (0.69, 2.02)** | 0.27 (0.02, 3.86) | 0.00 (0.00, 0.00) | 0.75 (0.30, 1.89) | 0.64 (0.27, 1.56) | 0.18 (0.02, 1.48) | 0.00 (0.00, 0.00) | 0.01 (0.00, 1.42) |
| BMI |  |  |  |  |  |  |  |  |
| Normal | **1.39 (1.33, 1.45)** | **0.72 (0.65, 0.80)** | 1.05 (0.96, 1.14) | **1.33 (1.25, 1.42)** | **1.11 (1.03, 1.18)** | **1.08 (1.00, 1.17)** | **1.15 (1.06, 1.26)** | 0.98 (0.90, 1.06) |
| Underweight | **1.50 (1.33, 1.70)** | **0.37 (0.21, 0.68)** | 0.90 (0.68, 1.18) | **1.51 (1.22, 1.87)** | 1.08 (0.84, 1.37) | 0.93 (0.68, 1.27) | 1.03 (0.77, 1.39) | 0.88 (0.67, 1.17) |
| Overweight | **1.39 (1.31, 1.47)** | **0.71 (0.62, 0.81)** | 1.01 (0.91, 1.13) | **1.32 (1.21, 1.44)** | 1.10 (1.00, 1.21) | 1.05 (0.94, 1.17) | 1.12 (1.00, 1.26) | 0.96 (0.86, 1.07) |
| Obesity | **1.47 (1.25, 1.74)** | 0.93 (0.68, 1.27) | 0.90 (0.65, 1.24) | 1.31 (0.97, 1.76) | 1.00 (0.74, 1.35) | 0.90 (0.63, 1.30) | 1.06 (0.75, 1.52) | 0.87 (0.62, 1.23) |

Model adjusted for age, education, BMI, annual household income, current smoking status, current alcohol consumption status, current tea consumption, medical history of stroke, diabetes, hypertension, cancer, dyslipidemia, current vegetable consumption, current fruit consumption, current seafood consumption, current freshwater product consumption, current marine product consumption, current meat consumption, current red meat consumption, and current white meat consumption.

Table S11**.** Sensitive analysis of association between long term exposure to metal and nonmetal elements and risk of depression and anxiety onset

|  | Depression (603 cases/108318.49 person years) | | |  | Anxiety (1102 cases/110016.61 person years) | | |
| --- | --- | --- | --- | --- | --- | --- | --- |
|  | Model 1 | Model 2 | Model 3 |  | Model 1 | Model 2 | Model 3 |
| Manganese | 0.97 (0.90, 1.05) | 0.98 (0.90, 1.06) | 0.96 (0.88, 1.04) |  | 1.41 (1.36, 1.45) | 1.41 (1.37, 1.46) | 1.39 (1.34, 1.45) |
| Zinc | 1.06 (0.97, 1.15) | 0.99 (0.90, 1.08) | 1.00 (0.92, 1.09) |  | 0.70 (0.64, 0.77) | 0.66 (0.60, 0.72) | 0.71 (0.64, 0.77) |
| Copper | 1.25 (1.15, 1.36) | 1.15 (1.02, 1.29) | 1.14 (1.01, 1.28) |  | 1.15 (1.09, 1.22) | 1.05 (0.98, 1.14) | 1.03 (0.95, 1.11) |
| Iron | 1.08 (1.02, 1.16) | 1.03 (0.95, 1.11) | 0.99 (0.92, 1.08) |  | 1.41 (1.34, 1.49) | 1.41 (1.34, 1.49) | 1.34 (1.26, 1.42) |
| Aluminum | 1.19 (1.12, 1.27) | 1.14 (1.05, 1.22) | 1.12 (1.04, 1.21) |  | 1.21 (1.16, 1.27) | 1.17 (1.11, 1.24) | 1.10 (1.04, 1.18) |
| Cadmium | 1.22 (1.14, 1.30) | 1.14 (1.06, 1.24) | 1.13 (1.03, 1.23) |  | 1.18 (1.12, 1.25) | 1.12 (1.04, 1.20) | 1.07 (0.99, 1.15) |
| Selenium | 1.18 (1.09, 1.28) | 1.08 (0.97, 1.19) | 1.07 (0.96, 1.19) |  | 1.23 (1.15, 1.31) | 1.19 (1.09, 1.29) | 1.15 (1.05, 1.25) |
| Fluorine | 1.16 (1.07, 1.25) | 1.04 (0.94, 1.15) | 1.08 (0.97, 1.19) |  | 1.06 (1.00, 1.12) | 0.95 (0.88, 1.02) | 0.99 (0.91, 1.06) |

Age and education were adjusted in Model 1. Annual household income, BMI, current smoking status, current tea consumption, current alcohol consumption status, medical history of stroke, diabetes, hypertension, cancer, and dyslipidemia were further adjusted in Model 2. Current fruit consumption, current vegetable consumption, current freshwater product consumption, current marine product consumption, current seafood consumption, current red meat consumption, current white meat consumption, and current meat consumption were further adjusted in Model 3.

Table S12. E-value for point estimates and the lower 95% confidence intervals of the Hazard

|  | Depression | | |  | Anxiety | | |
| --- | --- | --- | --- | --- | --- | --- | --- |
|  | Model 1 | Model 2 | Model 3 |  | Model 1 | Model 2 | Model 3 |
| Manganese | 0.94 (0.84, 1.05) | 0.95 (0.88, 1.03) | 0.92 (0.88, 0.97) |  | 1.42 (1.20, 1.67) | 1.43 (1.20, 1.70) | 1.39 (1.20, 1.62) |
|  | 1.32 (1.00) | 1.29 (1.00) | 1.39 (1.21) |  | 2.19 (1.69) | 2.21 (1.69) | 2.13 (1.69) |
| Zinc | 1.00 (0.85, 1.18) | 0.93 (0.77, 1.12) | 0.95 (0.84, 1.08) |  | 0.70 (0.45, 1.09) | 0.66 (0.42, 1.03) | 0.71 (0.47, 1.08) |
|  | 1.00 (1.00) | 1.36 (1.00) | 1.29 (1.00) |  | 2.21 (1.00) | 2.40 (1.00) | 2.17 (1.00) |
| Copper | 1.18 (1.13, 1.24) | 1.07 (1.02, 1.12) | 1.06 (1.01, 1.11) |  | 1.15 (1.06, 1.26) | 1.05 (0.98, 1.13) | 1.02 (0.97, 1.08) |
|  | 1.64 (1.51) | 1.34 (1.16) | 1.31 (1.11) |  | 1.57 (1.31) | 1.28 (1.00) | 1.16 (1.00) |
| Iron | 1.08 (0.91, 1.27) | 1.03 (0.89, 1.20) | 0.99 (0.89, 1.10) |  | 1.41 (1.15, 1.72) | 1.41 (1.11, 1.81) | 1.33 (1.03, 1.71) |
|  | 1.37 (1.00) | 1.21 (1.00) | 1.11 (1.00) |  | 2.17 (1.57) | 2.17 (1.46) | 1.99 (1.21) |
| Aluminum | 1.15 (0.99, 1.33) | 1.10 (0.93, 1.30) | 1.08 (0.93, 1.26) |  | 1.22 (1.04, 1.42) | 1.17 (0.95, 1.44) | 1.10 (0.92, 1.31) |
|  | 1.57 (1.00) | 1.43 (1.00) | 1.37 (1.00) |  | 1.74 (1.24) | 1.62 (1.00) | 1.43 (1.00) |
| Cadmium | 1.17 (1.03, 1.33) | 1.10 (0.98, 1.22) | 1.08 (1.00, 1.17) |  | 1.18 (0.94, 1.48) | 1.11 (0.86, 1.44) | 1.06 (0.89, 1.26) |
|  | 1.62 (1.21) | 1.43 (1.00) | 1.37 (1.00) |  | 1.64 (1.00) | 1.46 (1.00) | 1.31 (1.00) |
| Selenium | 1.14 (1.06, 1.22) | 1.04 (0.99, 1.08) | 1.03 (0.99, 1.06) |  | 1.22 (1.10, 1.35) | 1.18 (1.08, 1.28) | 1.14 (1.03, 1.25) |
|  | 1.54 (1.31) | 1.24 (1.00) | 1.21 (1.00) |  | 1.74 (1.43) | 1.64 (1.37) | 1.54 (1.21) |
| Fluorine | 1.09 (1.00, 1.19) | 0.97 (0.86, 1.09) | 1.00 (0.91, 1.09) |  | 1.04 (0.88, 1.24) | 0.92 (0.72, 1.17) | 0.96 (0.78, 1.19) |
|  | 1.40 (1.00) | 1.21 (1.00) | 1.00 (1.00) |  | 1.24 (1.00) | 1.39 (1.00) | 1.25 (1.00) |

Age and education were adjusted in Model 1. Annual household income, BMI, current smoking status, current tea consumption, current alcohol consumption status, medical history of stroke, diabetes, hypertension, cancer, and dyslipidemia were further adjusted in Model 2. Current fruit consumption, current vegetable consumption, current freshwater product consumption, current marine product consumption, current seafood consumption, current red meat consumption, current white meat consumption, and current meat consumption were further adjusted in Model 3.


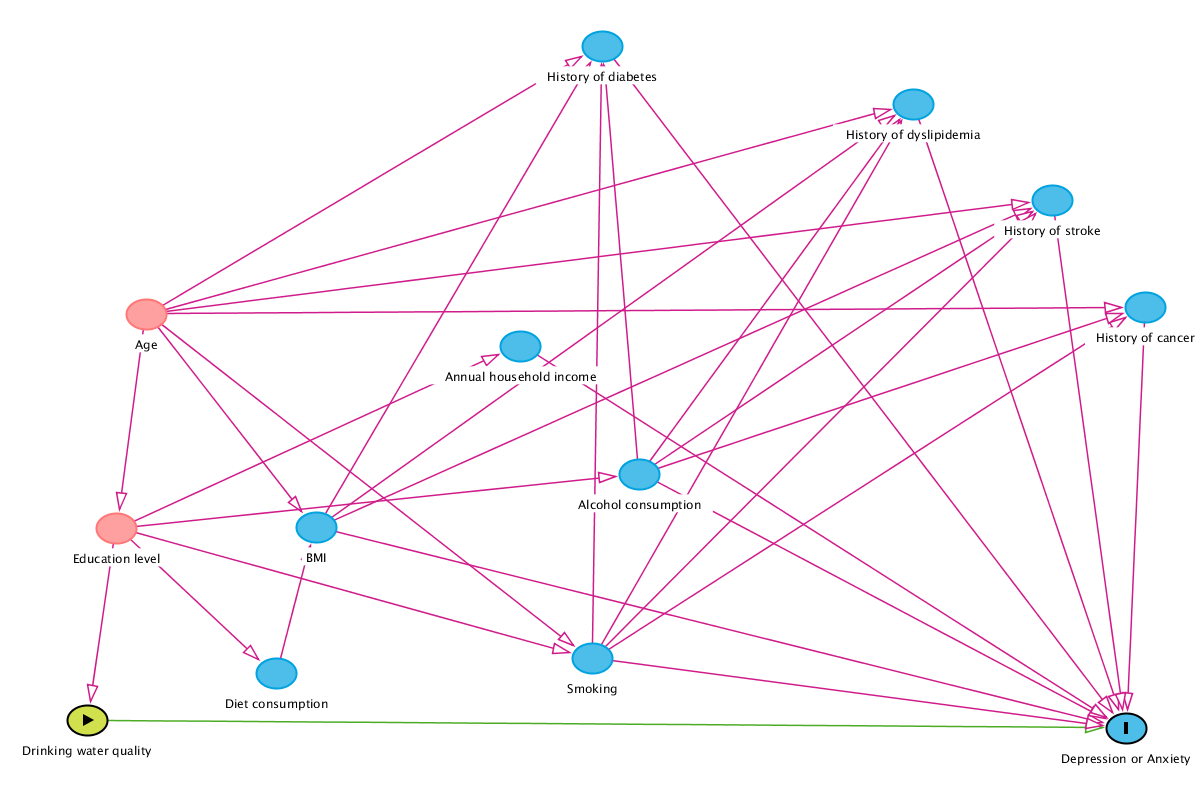


Figure S1. Directed acyclic graph for water exposure and depression, anxiety

derived directly from <http://www.dagitty.net/dags.html>.

Green circle with triangle: exposure; green circle: ancestor of exposure; blue circle with stop sign: outcome; blue circle: ancestor of outcome; red circle: ancestor of exposure and outcome.
